# Evolution of weedy giant ragweed (*Ambrosia trifida*): Multiple origins and gene expression variability facilitates weediness

**DOI:** 10.1002/ece3.9590

**Published:** 2022-12-08

**Authors:** Bo Li, Andrea R. Gschwend, Stephen M. Hovick, Amanda Gutek, Leah McHale, S. Kent Harrison, Emilie E. Regnier

**Affiliations:** ^1^ Department of Horticulture and Crop Sciences The Ohio State University Columbus Ohio USA; ^2^ Department of Evolution, Ecology and Organismal Biology The Ohio State University Columbus Ohio USA

**Keywords:** adaptation, *Ambrosia trifida*, differential gene expression, giant ragweed, RNA‐seq, weed population structure

## Abstract

Agricultural weeds may originate from wild populations, but the origination patterns and genetics underlying this transition remain largely unknown. Analysis of weedy‐wild paired populations from independent locations may provide evidence to identify key genetic variation contributing to this adaptive shift. We performed genetic variation and expression analyses on transcriptome data from 67 giant ragweed samples collected from different locations in Ohio, Iowa, and Minnesota and found geographically separated weedy populations likely originated independently from their adjacent wild populations, but subsequent spreading of weedy populations also occurred locally. By using eight closely related weedy‐wild paired populations, we identified thousands of unique transcripts in weedy populations that reflect shared or specific functions corresponding, respectively, to both convergently evolved and population‐specific weediness processes. In addition, differential expression of specific groups of genes was detected between weedy and wild giant ragweed populations using gene expression diversity and gene co‐expression network analyses. Our study suggests an integrated route of weedy giant ragweed origination, consisting of independent origination combined with the subsequent spreading of certain weedy populations, and provides several lines of evidence to support the hypothesis that gene expression variability plays a key role in the evolution of weedy species.

## Graphical abstract

We investigated the population genetic structure and expression patterns of giant ragweed plants sourced from crop and noncrop habitats across the Eastern Corn Belt of the USA, where this native species appears to have undergone one or more evolutionary transitions to succeed in crop fields as a major agricultural weed. The results of our study suggest an integrated route of weedy giant ragweed origination, consisting of independent origination combined with the subsequent spreading of certain weedy populations, and provide several lines of evidence to support the hypothesis that gene expression variability plays a key role in the evolution of weedy species.

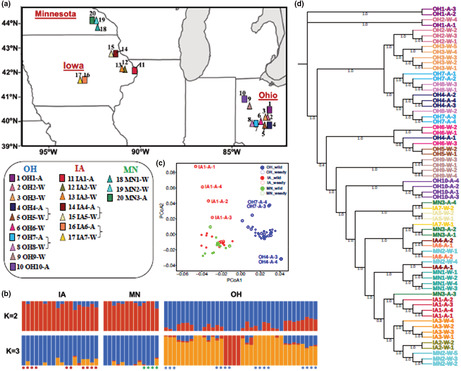

## 1 INTRODUCTION

The evolution of agricultural weeds generally occurs within a short time frame, thus making agricultural weeds an ideal system for understanding adaptive evolution in plants (Guo et al., 2018). Agricultural weeds can originate through various routes, including (1) invasion of crop fields by wild plants; (2) hybridization (between two wild species or between a crop and wild species); or (3) de‐domestication of a crop species (Kane & Rieseberg, 2008; Vigueira et al., 2013). Here, we focus on the first of these pathways, in which weeds originate from wild populations by adapting rapidly, undergoing changes in growth rate, phenology, herbicide tolerance, and resistance to environmental stress (Basu et al., 2004; Hovick et al., 2018; van Boheemen et al., 2019). Agricultural practices result in dramatic changes to long‐term selective pressures on standing variation for newly adaptive traits and novel mutations that improve fitness in new environments. Yet, rapid adaptation need not only reflect genetic changes within genes, because changes in gene expression can also be adaptive (Kane & Rieseberg, 2008; Lai et al., 2008; Mayrose et al., 2011). The recent, rapid emergence of several North American native dicots as major agricultural weeds in their native range (e.g., species of *Ambrosia, Helianthus* and *Amaranthus*) raises questions as to whether weedy populations tend to originate from multiple, independent wild populations or from a single origin, whether the transition to weediness across a species' range has a common genetic basis, and whether transcriptome changes hasten the evolution of weediness (Bock et al., 2020; Davis et al., 2015; Jhala et al., 2014; Leon et al., 2021; Regnier et al., 2016; Sauer, 1957; Tranel & Trucco, 2009; Ward et al., 2013; Waselkov et al., 2020).

Giant ragweed (*Ambrosia trifida*) is an annual dicot that grows in open, disturbed, and ruderal habitats and exhibits substantial phenotypic variability, both within and among populations. Its large population sizes and outcrossing mating system may also increase genetic variance and adaptation in response to natural selection. Beyond sharing these characteristics with other North American native weeds, giant ragweed is well‐suited to investigate the drivers of repeated invasion of crop fields by wild species because it is widely distributed among diverse early‐successional habitats, yet varies geographically in its presence and severity as an agricultural weed (Regnier et al., 2016). Specifically, despite its presence as an important agricultural weed for decades in the Eastern Corn Belt, giant ragweed has only relatively recently spread to agricultural settings in the western part of its native range (Regnier et al., 2016). This variation in weediness, set against a near‐continuous background of wild populations, affords an excellent opportunity to compare wild and weedy populations and to test hypotheses of weed origins and adaptation.

Although genetic differences between agricultural weeds and their wild relatives have been documented, closely related weedy and wild populations have rarely been compared (Ellstrand et al., 2010). Identifying the genetic processes involved in the evolution of agricultural weeds from wild relatives requires identifying progenitor populations and characterizing genetic differences between progenitor and weedy populations (Guo et al., 2018). It may be difficult to trace the origin of a weedy population, particularly in outcrossing species that hybridize frequently and where weedy populations migrated far from their progenitor population. In addition, weedy populations at different locations may evolve similar or different traits, depending on local selection pressures, novel mutations, gene flow, drift, and the genetic backgrounds of progenitor populations (Délye, Menchari, et al., 2013; Ghanizadeh et al., 2019; Huang et al., 2017; Kane & Rieseberg, 2008; Vigueira et al., 2013). Weedy giant ragweed is characterized by substantial morphological and genetic variability within and among populations, including the agriculturally important adaptations of herbicide tolerance, as well as seedling emergence that is both delayed and prolonged relative to nearby wild populations (Hovick et al., 2018; Patzoldt & Tranel, 2002; Schutte et al., 2008). Given the strong selective pressures that herbicide application and early‐season weed management impose on weed populations, such traits are highly likely to be adaptive in agricultural fields but not in wild populations, thus contributing to these observed differences. Comparative analysis of weedy giant ragweed populations from multiple, distant locations enables testing for whether these, and other trait‐based signatures of weediness, have evolved from a single, or multiple, independent location(s) and whether adaptation to agricultural fields involves a common set of genes (Stewart et al., 2009).

While novel mutations in coding sequences can have adaptive properties, gene expression variability may also contribute to rapid adaptation in new environments, enabling population establishment and the postestablishment evolution of adaptive traits (Charbonneau et al., 2018; Fraser, 2013; Kane & Rieseberg, 2008; Lai et al., 2008; Mayrose et al., 2011; Vigueira et al., 2013). In fact, many genes are expressed differentially by weeds and their wild relatives, suggesting gene expression reprogramming may be critical for this evolutionary process (Lai et al., 2008; Leslie & Baucom, 2014; Mayrose et al., 2011; Xu et al., 2016). For example, gene expression changes have been observed in biotic and abiotic stimulus–response and stress‐related protein genes in weedy compared with wild sunflower populations (Lai et al., 2008; Mayrose et al., 2011) and in response to herbicide application in morning glory (*Ipomoea purpurea*; Josephs et al., 2021). Investigating differential gene expression between giant ragweed populations may help identify potential mechanisms by which this transition from wild to weedy occurred.

To understand the origins of agricultural weedy populations of giant ragweed (which lacks a sequenced genome) and the genetics distinguishing weedy from wild populations, we conducted RNA‐sequencing for 67 samples collected from 20 giant ragweed populations growing across the east‐central U.S. Corn Belt, either in nonagricultural wild habitats or in crop fields (weedy populations). We studied population structure, identified the most closely related wild populations to each of our weedy populations, and uncovered gene expression variation that distinguished weedy from wild populations. Our study suggests weedy giant ragweed populations arose via multiple independent origination events from local wild populations, followed by the spread of weedy populations regionally across crop fields. Differentially expressed genes, especially those involved in seed germination, vegetative stage change, and abiotic stress responses, may contribute to weediness in giant ragweed. Our results support the hypothesis that gene expression variability plays a key role in the convergent evolution of weedy plants.

## 2 MATERIALS AND METHODS

### 2.1 Sampling strategy

**FIGURE 1:**
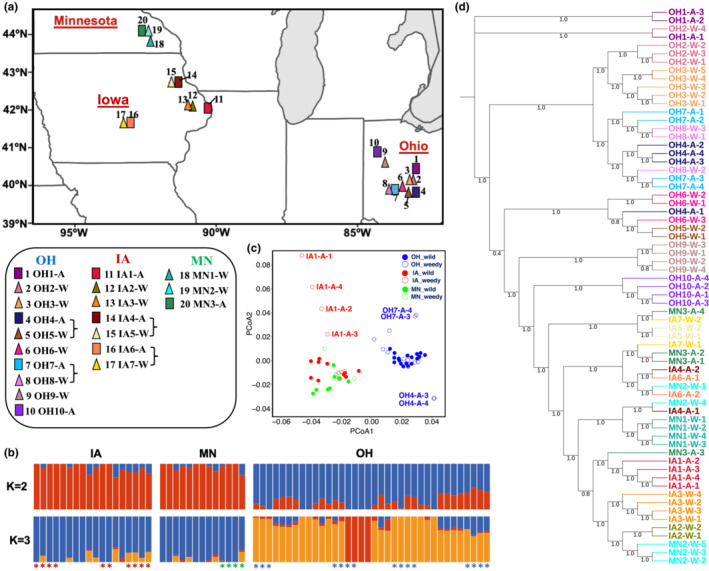
Population structure and phylogenetic analysis of giant ragweed populations. (a) Geographic locations of the 20 giant ragweed populations sampled. Colored rectangles indicate weedy (designated with an “A” for agricultural) and triangles indicate wild populations (designated with a “W”), respectively. Brackets connect geographically adjacent populations. (b) Ancestry analysis using ADMIXTURE with *K* = 2 and *K* = 3. Asterisks indicate samples from weedy populations and are color coded by state. (c) PCoA analysis with all samples. (d) Phylogeny of all samples inferred by the entire SNP dataset. Color code is consistent with the map (a).

Seeds from individual giant ragweed plants were collected in the east‐central U.S. Corn Belt (*n* = 20 populations) in fall 2011 and stored at 4°C until planting. Seeds from a total of 20 populations (2–5 maternal plants per population for a total of 67 samples) were collected from two regions, Ohio and Iowa‐Minnesota (Figure 1a). Within each region, we collected four weedy populations from agricultural (corn or soybean) fields and six wild populations from nonagricultural habitats (e.g., riverbanks and fencerows). Among them, samples from four pairs of weedy and wild populations were collected as paired adjacent populations sourced from the same geographic location (i.e., within 100 meters of each other). Otherwise, all populations were at least 2.7 kilometers apart. All populations were named with their state abbreviation (OH, IA, or MN), numeric order according to longitude from east to west, habitat (A for weedy populations in agricultural fields and W for wild, nonagricultural populations), and finally each individual sample was designated with a unique trailing numeral, for example, OH1‐A‐1 (Table S1).

### 2.2 RNA extraction and sequencing

Seeds from 67 samples were planted and grown under standardized greenhouse conditions for tissue collection. The fourth set of true leaves from each plant was collected and total RNA extracted using NucleoSpin RNA Plant kits (Macherey‐Nagel) following manufacturer's instructions. RNA‐seq libraries were prepared using the Illumina TruSeq Stranded mRNA Library Prep Kit (Illumina, Inc.), with library quality subsequently analyzed by qPCR using KAPA Library Quantification kits (Kapa Biosystems, Inc.), Qubit 2.0 Fluorometric (Invitrogen, Life Technologies), and a 2100 Bioanalyzer. Sample libraries were pooled according to the sequencing instrument's requirements and sequenced using Illumina HiSeq2500.

### 2.3 Reference transcriptome assembly and evaluation

We established a comprehensive pipeline for transcriptome construction, variant calling, and differential expression analysis with our samples (Figure S1). We randomly selected a wild giant ragweed sample from an OH wild population (OH6‐W‐3) and generated 47 M paired‐end reads to build a giant ragweed reference transcriptome. Raw read quality was evaluated with FastQC (Andrews, 2010), trimmed by Trimmomatic (Bolger et al., 2014), and de novo assembled with Trinity v2.9.0 using default parameters (Grabherr et al., 2011). We evaluated the assembled transcript quality using several methods: (1) We first mapped RNA‐seq data onto the reference transcripts using Bowtie 2 (Langmead & Salzberg, 2012) to test RNA‐seq read representation of transcripts (overall mapping rate of 98.45%). (2) We used BUSCO genes to assess transcript coverage of orthologous genes (Seppey et al., 2019). (3) We evaluated the completeness of our transcripts by aligning them to the Uniprot database (https://www.uniprot.org/) and to cDNA data from closely related, *Helianthus annuus* (https://plants.ensembl.org), since a reference genome and gene annotation for A*mbrosia trifida* is not available. We removed redundant transcripts by applying CD‐HIT to cluster transcripts with >98% similarity (Fu et al., 2012), predicted transcript coding regions with TransDecoder v5.5.0 (Haas et al., 2013), and functionally annotated the predicted protein sequences with InterProScan (Jones et al., 2014). We also quantified transcript expression, defining transcript ≥1 TPM (transcript per kilobase per million reads) as expression support. In the end, all assembled transcripts lacking either predicted protein domains or expression support were removed to establish a filtered transcriptome.

### 2.4 RNA‐seq‐based variant calling

To call single‐nucleotide polymorphisms (SNP), we combined GATK RNA‐seq best practices with Joint genotyping (Brouard et al., 2019; Van der Auwera et al., 2013). RNA‐seq data from all 67 ragweed samples were mapped onto the filtered reference transcriptome with STAR in a two‐pass model (‐‐twopassMode Basic) (Dobin et al., 2013). Then, GATK4 was used to call variants for each sample according to RNA‐seq best practice. To remove false SNPs, we compared SNP calls between two biological replicates (RNA extracted from the same plant), removing SNPs not found in both samples. This final step also removed putative tri‐allelic variants resulting from paralog misalignment.

### 2.5 Population genetic diversity and genetic structure analyses

Geographic distances between each pair of populations were calculated using latitudes and longitudes. Fixation indices (*F*_ST_) among populations were calculated using VCFtools (Danecek et al., 2011)with –weir‐fst‐pop parameters (Weir & Cockerham, 1984). Mantel tests (R package *ade4*) were performed to correlate pairwise *F*_ST_ versus geographical distances and test for isolation by distance within region; these tests did not encompass all populations in a single analysis because doing so violates model assumptions. We calculated observed and expected heterozygosity (*H*_o_ and *H*_e_) and nucleotide diversity (π) per population using VCFtools.

Population structure was inferred based on filtered SNP data with ADMIXTURE (Alexander et al., 2009). From *K* = 1 to *K* = 7, we calculated and compared cross‐validation error to choose the best ancestral population inference. PCoA analysis was performed with PLINK 1.9 (Purcell et al., 2007). FastTree was used to build approximately maximum‐likelihood phylogenetic trees (Price et al., 2010). Trees were displayed and modified with iTOL (Letunic & Bork, 2019). To check that inferences were not driven by SNPs under selection, we also built trees using SNPs that occurred in third‐codon positions and were synonymous for amino acid identity; results were qualitatively the same, so we present analyses using the full dataset.

### 2.6 Gene expression diversity between weedy and wild populations

RNA‐seq data were mapped onto our reference transcriptome by HiSAT2 (Kim et al., 2019). Normalized expression values (transcripts per kb per million, or TPM) were calculated with StringTie (Pertea et al., 2015). We calculated the coefficient of variation (CV) for transcript abundance in each population to represent gene expression diversity (GED; Bellucci et al., 2014), which we interpreted as an indicator of genetic variability. We first compared GED between all weedy versus all wild samples using Kruskal–Wallis tests, doing so separately for OH and IA_MN populations. We extracted the transcript IDs under each shifted peak region and conducted GO annotation for functional inference. Then, all eight weedy populations were paired with the most genetically similar wild population and population‐level GED was compared. We equalized sample sizes for these weedy‐wild pairs by randomly downsizing larger populations before calculating GED. We extracted transcript IDs that had more than twofold difference in GED (CV values) in weedy populations compared with wild populations and conducted functional annotation on these gene groups. GO enrichment analysis was done with ClusterProfiler (Yu et al., 2012), and the adjusted *p*‐value cutoff was .05 (Benjamini–Hochberg method).

### 2.7 Differential gene expression analysis

We conducted differential gene expression analysis by estimating raw read counts for each gene by HTSeq based on mapping results (Anders et al., 2015). We identified differentially expressed genes between paired weedy and wild populations using DESeq2 (Love et al., 2014). To functionally annotate differentially expressed genes, we aligned our giant ragweed protein sequences onto the *Uniprot* dataset and then linked the Gene Ontology (GO) term and function description to aligned giant ragweed proteins to create a giant ragweed GO annotation file. Differentially expressed genes were subjected to functional enrichment analysis using ClusterProfiler.

### 2.8 De novo assembly of unmapped reads from each population

Our giant ragweed reference transcriptome was established using one wild sample (described above); thus, transcripts unique to (i.e., only expressed in) other samples, like weedy populations, would be absent from the reference sequence. To recover these unique transcripts, we collected RNA‐seq reads that were unmapped after alignment to the reference transcriptome. For each population, unmapped reads from individuals were collected for de novo transcript assembly. The transcripts from each weedy population were then used as references for mapping RNA‐seq reads from wild samples to filter out the weedy‐wild shared transcripts and vice versa. The unmapped transcripts were collected as unique transcripts for each weedy or wild population. These unique transcripts were then used to predict open reading frames (ORFs), removing transcripts without predicted protein‐coding ability from further analysis. Unique transcripts showing sequence similarity from different populations were identified using OrthoVenn2 with cutoff as 1e^−5^ (Xu et al., 2019).

### 2.9 Co‐expression gene network analysis

Two independent co‐expression gene networks were established for weedy and wild populations. We filtered out transcripts with low expression (TPM ≤ 1 in >80% of all samples). Then, gene networks were constructed using WGCNA (Langfelder & Horvath, 2008). The soft power parameter was 6 for both weedy and wild samples. Only modules with more than 30 genes were maintained. To identify conserved and variable modules between weedy and wild samples, we related all modules identified in weedy samples to wild samples by calculating gene overlap of each paired module. Significant overlap was estimated using Fisher's exact test, with *p*‐values transformed into −log_10_(*p*). Functional annotations were conducted on the most conserved and variable modules with ClusterProfiler.

### 2.10 Herbicide resistance genes analysis

cDNA sequences of seven giant ragweed herbicide resistance genes (5‐enolpyruvylshikimate‐3‐phosphate synthase (*EPSPS*), glutamine synthetase (*GS*), acetyl‐coenzyme A carboxylase (*ACCase*), acetolactate synthase (*ALS*), 4‐hydroxyphenylpyruvate dioxygenase (*HPPD*), phytoene desaturase (*PDS*), and protoporphyrinogen oxidase (*PPO*)) were retrieved from the International Survey of Herbicide Resistant Weeds (http://www.weedscience.org). BLASTn was used to search the full giant ragweed transcriptome with these cDNA sequences as queries (Altschul et al., 1997). Only hits with 90% identity and 90% coverage were maintained as corresponding to herbicide resistance genes. TPM for each gene was extracted from our gene expression dataset, logarithm‐transformed, and compared between weedy and wild populations to infer pseudo copy number amplifications. For each gene, we gathered all SNPs discovered across the transcript and compared allele frequencies between weedy and wild populations. For each SNP, Fisher's exact test was used to identify sites with significantly different allele frequencies. For the *ALS* gene, we identified all missense mutations and recorded the positions and amino acid changes. We then compared giant ragweed and *Arabidopsis thaliana* ALS protein sequences, using Clustal Omega, to confirm the occurrence of previously identified *ALS* mutations in giant ragweed conferring resistance to ALS‐inhibiting herbicides (Sievers et al., 2011).

## 3 RESULTS

### 3.1 RNA sequencing, transcriptome assembly, and variant calling

Sixty‐seven RNA‐seq datasets were generated from 20 populations (Figure 1a and Table S1), and one deeply sequenced sample was used to establish a giant ragweed reference transcriptome, composed of 41,669 trinity genes or 91,296 transcripts (Table S2). BUSCO assessment showed coverage of 91.3% conserved eukaryote ortholog genes with only 89 missing gene models (Figure S2). About 57.2% of transcripts had protein homologs in the *Uniprot* database. Overall, 616,607 SNPs from the 67 giant ragweed transcriptomes were identified and used for downstream analyses (Table S3).

### 3.2 Population analyses suggest independent origins with local spreading of weedy giant ragweed populations

Based on SNP data, we estimated the overall transcriptome nucleotide diversity (*π*), averaged across all giant ragweed populations, to be 0.0024. Nucleotide diversity did not differ between weedy and wild populations (Wilcoxon signed‐rank test, *p* = .77; Table S4). Observed heterozygosity (*H*_o_) was lower than expected heterozygosity (*H*_e_) for all but one population (Table S4).

ADMIXTURE results revealed the inference of a single ancestral population (*K* = 1) had the smallest cross‐validation error (CVE = 0.5323; Figure S3), but the inference of two ancestral populations was only slightly less favored (CVE = 0.5413) and separated our two geographic regions into distinct subpopulations: OH and combined IA and MN samples (hereafter IA‐MN; Figure 1b). Principal coordinates analysis (PCoA) further indicated that OH populations were distinct from IA‐MN populations, which cluster together (Figure 1c). According to ADMIXTURE population structure analyses, individuals from weedy populations tended to be more genetically similar to wild individuals from populations that were geographically close (versus being similar to other weedy populations despite their geographic location; Figure 1b), suggesting that weedy populations in our study originated from multiple distinct ancestor populations.

To further infer the origins of weedy populations, we established a phylogenetic tree based on the full transcriptome SNP dataset (Figure 1d). All samples from OH were completely separated from IA‐MN populations, consistent with population structure analyses (Figure 1b,c). Based on our PCoA results, we hypothesized that wild and weedy giant ragweed populations that were found geographically close to each other would also be genetically closely related. For most weedy populations within the OH subgroup, the geographically closest wild population was the most genetically similar (e.g., clustering OH1‐A with OH2‐W, OH7‐A with OH8‐W, and OH10‐A with OH9‐W; see Figure 1d), suggesting these weedy populations originated from nearby wild giant ragweed populations. However, for weedy population OH4‐A, the most genetically similar wild population was OH8‐W, rather than its geographically proximate wild population OH5‐W (Figure 1a,d), indicating that weedy OH4‐A may have instead originated from OH8‐W or been directly derived from OH7‐A, or another weedy population, via movement among crop fields (Figure 1d). This conclusion of mixed origins is further supported by phylogenetic relationships among IA‐MN samples. Except for IA1‐A, which was most genetically similar to its closest wild populations, IA2‐W/IA3‐W, the other weedy populations showed discrepancies between geographical proximity and genetic similarity (Figure 1d), suggesting that widespread dispersal of weedy individuals may have occurred more commonly in this region, in tandem with independent origination.

**FIGURE 2:**
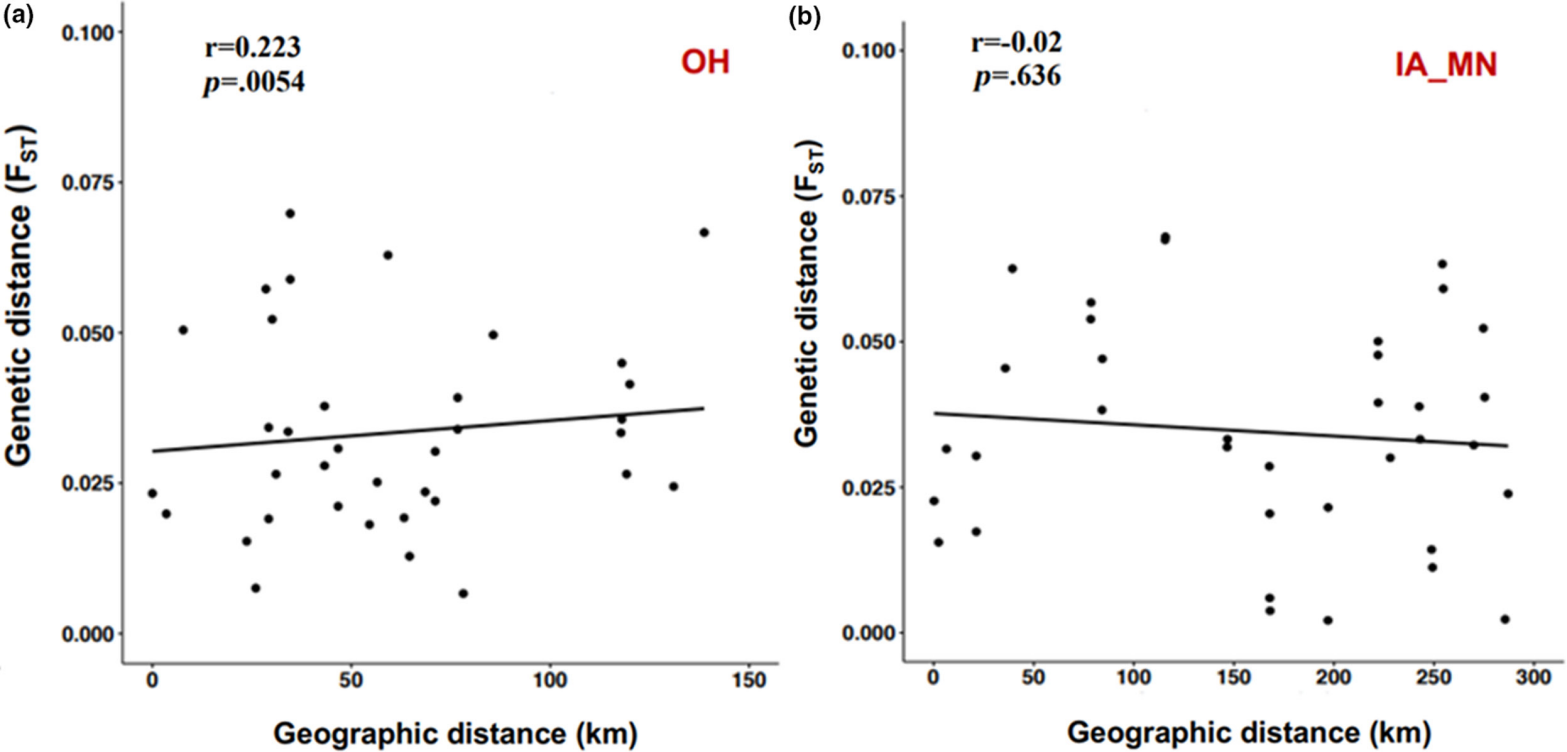
Isolation by distance analysis. Correlations between pairwise genetic distances (*F*_ST_) and geographic distances were assessed with Mantel tests across (a) the OH subgroup and (b) the IA‐MN subgroup populations. Best‐fit lines are shown for each dataset.

We tested the hypothesis of isolation by distance (IBD) among populations. The fixation index (*F*_ST_) among all population pairs was low (*F*_ST_ range = 0.002–0.109), suggesting that our giant ragweed populations were not greatly differentiated overall (Table S5). However, pairwise F_ST_ values were larger when comparing OH versus IA samples (0.059 ± 0.0023 [SEM]) and OH versus MN samples (0.0518 ± 0.0032), relative to IA versus MN (0.0277 ± 0.0035), indicating some degree of differentiation between OH versus IA‐MN populations. Mantel tests performed separately by region indicated that genetic distance was significantly correlated with geographical distance within OH (Figure 2a) but not within the IA‐MN region (Figure 2b). The lack of significant IBD for IA‐MN samples is in line with phylogenetic relationships (Figure 1d) that suggest gene flow among populations in that region is common.

### 3.3 Gene expression diversity and differential gene expression between weedy and wild populations

We next focused on understanding gene expression differences between weedy and wild populations. We observed high reproducibility for our RNA‐seq data (*R* ^2^ = 0.98 for two OH5‐W biological replicates; Figure S4a) and high pairwise correlations (Pearson *r* > .9) for expression patterns among all samples, with the highest similarity among samples from the same population (Figure S4b). Dendrogram clustering based on expression similarity (Figure S4c) does not show the same degree of geographical clustering as indicated by our SNP data (Figure 1d), suggesting gene expression patterns in giant ragweed are less reliable than genetic markers for determining population structure compared with other systems (e.g., Kryvokhyzha et al., 2016). Nor were weedy populations separated from wild populations in the dendrogram (Figure S4c), suggesting that weediness is not defined by major uniform transcriptome differences.

**FIGURE 3:**
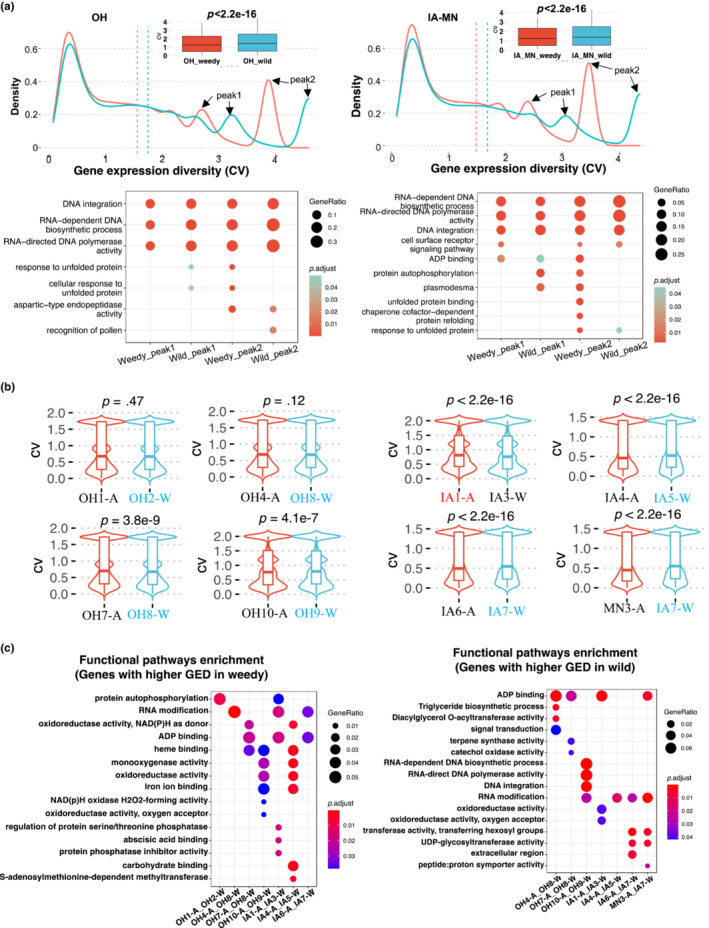
Gene expression diversity analysis. (a) Gene expression diversity (GED) was compared across all expressed genes between all weedy (red) and wild (blue) samples. Transcript density plots indicate CV distributions for weedy and wild samples, and vertical dashed lines represent mean values. Functional (GO) enrichment analyses were conducted for genes found in peak regions. (b) Pairwise GED comparisons among eight pairs of genetically similar weedy‐wild populations. Colored population names indicate the population with higher median GED, based on Wilcoxon tests. (c) Functional (GO) enrichment analysis for genes with increased GED in either weedy or wild populations from pairwise analyses. In panels (a) and (c), gene ratio is the number of genes in the test group divided by those in the background and P.adjust values account for FDR.

Gene expression diversity (GED) reflects the population‐level expression variability of every gene; genes with high GED have variable expression across samples, and genes with low GED have similar expression patterns across samples. We first compared GED between weedy and wild samples by region, finding significantly higher GED in wild populations than in weedy populations (Figure 3a). We noticed that there were two peak shifts between weedy and wild populations in both the OH and IA‐MN graphs in Figure 3a and conducted functional enrichment analyses on the genes with GED values contributing to the peaks. These peak regions were significantly enriched in genes with functions related to DNA integration, DNA biosynthetic process, and DNA polymerase activity (Figure 3a). Interestingly, these functional categories were consistent for both the OH and IA‐MN population groups, indicating some degree of consistency in which types of genes contribute to GED differences between weedy and wild populations (Figure 3a).

To better capture which genes contributed to the differences in GED, we performed weedy‐wild pairwise comparisons of expression diversity on eight genetically determined population pairs, based on our previous phylogenetic results (Figure 3b); note that the OH8‐W population was the most genetically similar wild population for two OH weedy populations (OH4‐A and OH7‐A). Across these pairs, we identified 887 to 2424 specific genes with > twofold higher GED in wild than in weedy populations and 1132 to 2303 genes with > twofold lower GED in weedy than in wild populations, representing functions including ADP binding, RNA modification, oxidoreductase activity, and 12 other pathways (Figure 3c); differential expression of genes involved in these biological pathways may therefore contribute to weedy adaptation. Interestingly, the annotated pathways (GOs) largely overlapped for sets of genes with higher GED in weedy and wild populations (Figure 3c), suggesting that genes within the same pathway can either increase or decrease in expression variability when weediness evolves.

**FIGURE 4:**
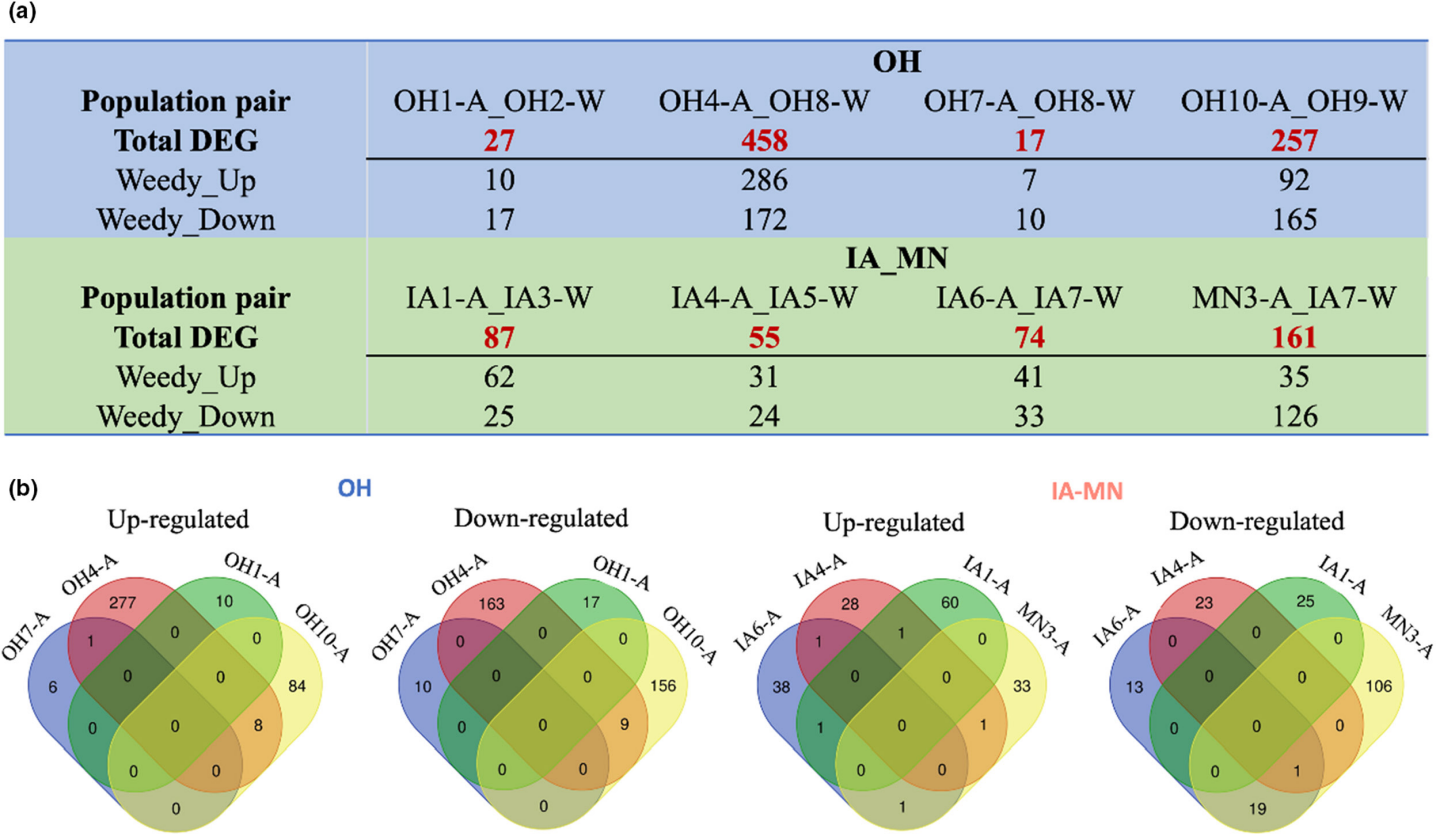
Differentially expressed gene (DEG) identification. (a) Differentially expressed genes identified within each weedy‐wild population pair. Both upregulated DEG (weedy‐up) and downregulated DEG (weedy‐down) in weedy populations were identified. (b) Overlap detection of upregulated and downregulated DEGs among weedy populations.

We identified between 17 and 458 differentially expressed genes (DEGs) across our eight weedy‐wild population pairs, with 21.7% to 71.3% upregulated in weedy populations (Figure 4a; Figures S5 and S6). However, very few (20 or less) DEGs were shared by multiple populations, suggesting most DEGs were population‐specific (Figure 4b). Functional annotation also showed that DEGs from different weedy populations were involved in distinct functional pathways, further indicating that our weedy populations did not share a common set of differentially expressed functional genes compared with our wild populations, at least under the conditions studied (Figure S7).

### 3.4 Unique transcripts in weedy populations may relate to adaptive traits

**FIGURE 5:**
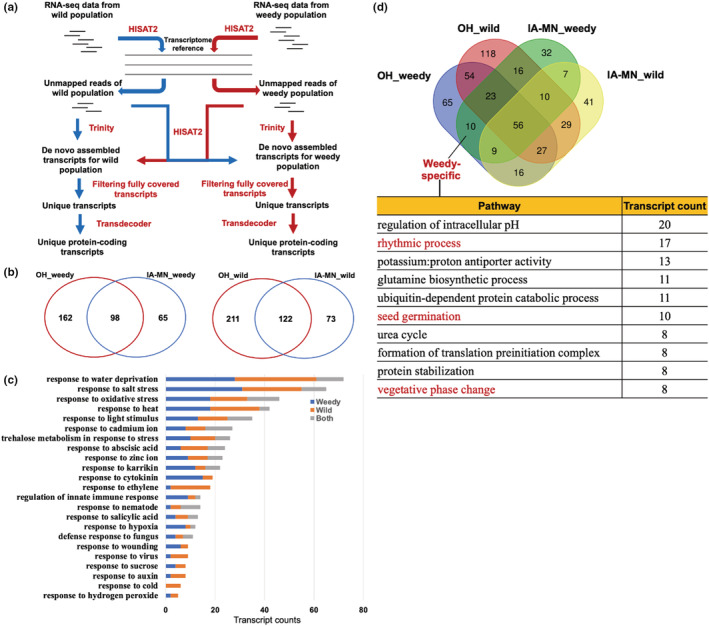
Unique transcripts in weedy populations. (a) Workflow for identifying unique transcripts between weedy and wild populations. (b) Overlap between functional categories annotated from commonly shared gene families containing unique transcripts between weedy and wild populations. (c) Weedy‐specific, wild‐specific, and orthologous (both) transcripts across the 23 shared functional categories. (d) Identification of specific functional groups in which unique weedy transcripts are involved, after removing all shared functions with wild samples.

To fully capture the identities of differentially expressed genes, we developed a novel pipeline to recover unique transcripts for each weedy‐wild population pair that could not be mapped to our reference transcriptome (see Section 2 and Figure 5a). For samples from the four OH weedy populations, RNA‐seq read mapping rates were between 69.75% and 80.09%, leaving ~0.4–2 M reads per sample for this de novo assembly (Table S6). From these four populations, we identified between 8537 and 23,492 transcripts present only in weedy populations and absent from the paired wild populations (Table S6). We applied an orthologous gene analysis approach on these unique transcripts and found 477 homologous gene groups (2317 genes) from all OH weedy populations (Figure S8). Functional annotation revealed these shared genes fell into 260 different functional categories (Table S7 and Figure S8). For IA‐MN weedy populations, we identified 4510 to 24,639 unique transcripts (Table S6) and 252 shared homologous gene groups (1301 genes), assigned to 163 functional groups (Figure S8). Further comparison of functional groupings revealed 98 groups (GO terms) shared among all OH and IA‐MN weedy populations (Figure 5b and Figure S8). These sets of unique transcripts indicate specific ways in which differential gene expression (inferred as the *presence* of unique transcripts) may contribute to the independent evolution of weediness across sites.

We repeated the above analysis with wild populations, completing de novo assembly using unmapped transcripts present only in our six wild populations and not in their paired weedy populations (Table S8). From this assembly, we characterized 787 homologous groups shared across OH (2640 genes in 333 functional groups) and 369 shared across IA‐MN (1257 genes in 195 functional groups; Figure S9). Wild populations from OH and those from IA‐MN shared 122 functional categories (Figure 5b and Figure S9). Of note, most of these response pathways also occured among transcripts unique to weedy populations, suggesting that both weedy and wild samples have unique transcripts expressed in similar pathways. Therefore, we further investigated whether the common pathways shared by weedy and wild giant ragweed also reflected similar genes. Despite 54.0% of the functional categories being shared between OH weedy and wild populations, a much lower proportion (22.5%) of total unique transcripts were identified as homologous genes between weedy and wild populations (Table S9). For example, across 23 pathways related to plant‐environment interactions, 213 and 211 specific transcripts were found in OH weedy and wild populations, respectively (Figure 5c), demonstrating that despite expressing transcripts that function in the same biological pathway, different transcripts are expressed in weedy and wild populations. However, 104 homologous gene groups (showing higher sequence similarity) were also identified between weedy and wild, suggesting some shared transcripts may not have been completely removed in our pipeline.

We further performed overlap detection with all weedy and wild functional groups and identified 10 weedy‐specific pathways, including seed germination, rhythmic process, vegetative phase change, which relates to seedling emergence timing, and pathways involved in nitrogen uptake and assimilation (urea cycle and glutamine biosynthesis; Figure 5d), suggesting the unique constitutive expression of transcripts involved in these pathways may play a critical role in weediness. An additional 65 and 32 unique biological pathways were found for weedy populations in OH and IA‐MN, respectively (Figure 5d). These groups of unique transcripts may be related to environment‐specific transcriptome reprogramming during the transformation from wild to weedy.

### 3.5 Gene networks between weedy and wild populations

We established independent co‐expression gene networks for weedy and wild samples by grouping together transcripts with similar expression profiles into modules, resulting in networks of 45 and 27 gene modules, respectively (Section 2 and Figure S10a). We then assessed module overlap between weedy and wild samples to identify modules with the same gene expression patterns (stable modules) and modules with different gene expression patterns (variable modules) between wild and weedy populations, finding 37 conserved modules (eight significantly overlapping (−log(*p*) ≥ 30) and 29 moderately overlapping (5 < −log(*p*) ≤ 30)) and eight variable modules (nonsignificantly overlapping, 0 ≤ −log(*p*) ≤ 5; Langfelder & Horvath, 2008; Figure S10b). Functional annotation revealed that gene modules with basic biological functions, such as ribosome biogenesis, DNA replication, and photosynthesis, tended to be more conserved than other functional groups (Table 1 and Figure S10c). The eight variable gene modules may indicate rewiring of gene expression during the transition from wild to weedy. Interestingly, we identified one variable gene module involved in the biosynthesis of branched‐chain amino acids, and another involved in xenobiotic transport (Table 1 and Figure S10c), both of which relate to herbicide resistance.

**TABLE 1:** Representative GO annotations of conserved and variable modules between weedy and wild co‐expression networks.

Conserved modules	Variable modules
Ribosome biogenesis	Autophagy
DNA replication	Branch‐chained amino acid biosynthetic process
Photosynthesis	Peptidyl‐serine phosphorylation
Calcium ion binding	Xenobiotic transport
Motor activity	Cellulose biosynthetic process
DNA repair	Transcription factor binding
Autophagy	Endoplasmic reticulum
	Golgi transport complex

### 3.6 Herbicide resistance gene identification

**FIGURE 6:**
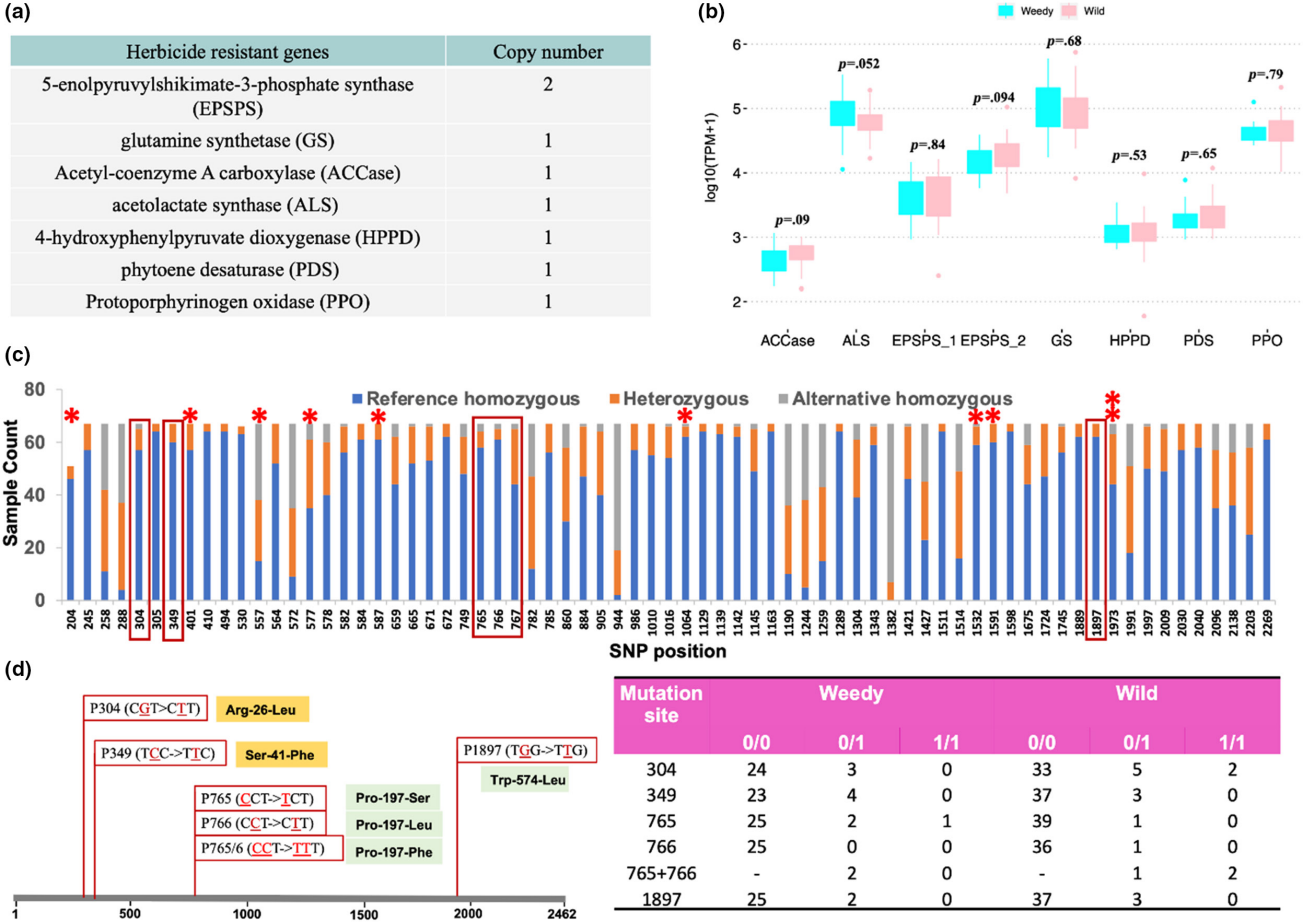
Herbicide resistance gene analysis. (a) Seven genes (eight transcripts) were characterized in the giant ragweed transcriptome. (b) Gene expression comparison between all weedy and wild samples (*p*‐value for each gene from Wilcoxon signed‐rank test). (c) SNP distribution across the ALS gene and genotype count across all samples. Three kinds of genotypes were assessed, and polymorphic sites with different allele frequency between weedy and wild are indicated with a red asterisk. Boxes indicate SNPs corresponding to nonconservative mutations. (d) Two previously confirmed resistant mutations (Pro‐197‐Leu (P766) and Trp‐574‐Leu (P1897)) (highlighted green) and two potential resistant mutations (Arg‐26‐Leu (P304) and Ser‐41‐Phe (P349)) (highlighted gold). The SNPs, codon change, and amino acid substitution are shown here. For example, P1897 represents a mutation from G to T at nucleotide position 1897 in the giant ragweed ALS transcript, which alters the codon from TGG to TTG, and subsequently replaces Tryptophan with Leucine at that location. Mutations P765 and P766 within one codon could cause three different kinds of amino acid replacements. Sample counts with different genotypes at these four positions are listed (0/0, reference homozygous; 0/1, heterozygous; and 1/1, alternative homozygous).

We tested for constitutive differential expression of transcripts for eight major herbicide‐target genes (Figure 6a) between weedy and wild samples to determine whether constitutive overexpression of these genes may contribute to weediness in giant ragweed. We did not detect significant differences in the herbicide resistance genes' expression between weedy and wild populations (Figure 6b), but the ALS gene did have borderline significant overexpression in weedy compared with wild samples (*p* = .052); thus, changes to the regulation or copy number of this gene in weedy individuals may have occurred.

Besides gene overexpression, changes in herbicide‐target gene coding sequences could also affect herbicide resistance. Several key mutations in genes coding for proteins targeted by herbicides have been identified that confer herbicide resistance (Tranel et al., 2018). Prior to this point, just one such mutation in ALS has been identified in both giant and common ragweed (a Trp‐574‐Leu substitution), which confers resistance to ALS inhibitors (Marion et al., 2017; Tranel et al., 2018). In our dataset, we characterized 71 SNPs across ALS transcripts and found six that caused nonconservative amino acid replacement at four loci (Figure 6c,d), including three ALS inhibitor resistance mutations that have been previously confirmed in multiple species (Pro‐197‐Ser, Pro‐197‐Leu, and Trp‐574‐Leu; Heap, 2021). We further identified a third type of mutation at codon 197, Pro‐197‐Phe, where two mutations (C > T at first‐codon position and C > T at second‐codon position) occur simultaneously, suggesting other resistance mutations may exist. We calculated allele frequencies for each SNP across the ALS transcripts and identified nine genotypes that were significantly different between weedy and wild populations. However, none of the nine segregating SNPs were found at the previously confirmed ALS inhibitor resistance loci (Figure 6d). Population genotyping analyses confirmed this observation, revealing that across 67 samples, two weedy and three wild samples had the Trp‐574‐Leu‐resistant genotype. Three weedy and one wild sample had the Pro‐197‐Ser substitution, one wild sample had the Pro‐197‐Leu substitution, and two weedy and three wild samples had the Pro‐197‐Phe substitution, implying ALS inhibitor‐resistant genotypes are not exclusive to the weedy populations we sampled (Figure 6d and Table S10).

For other herbicide‐target genes without experimentally confirmed herbicide resistance mutations in giant ragweed, we characterized all SNPs and identified polymorphic sites that significantly segregated between weedy and wild samples (Figure S11 and Table S11), providing a starting point for future studies to screen for novel mutations leading to herbicide resistance.

## 4 DISCUSSION

### 4.1 Giant ragweed transcriptome variability

We observed low nucleotide diversity among giant ragweed populations and found weedy and wild populations to be genetically similar within a given region, suggesting high gene flow across the sampled area. These results are consistent with giant ragweed's wind‐pollination mating system, in which outcrossing is expected to lead to high diversity within, but low diversity among, populations (Hovick et al., 2018; Leon et al., 2021; Radosevich et al., 2007). Limited genetic variation among populations has also been observed in other outcrossing native weeds, including common ragweed, sunflower, and water hemp (Hämälä et al., 2020; Kane & Rieseberg, 2008; Lai et al., 2008; McGoey & Stinchcombe, 2021; Waselkov et al., 2020). The use of transcriptome sequences in lieu of genome‐wide sequences may have contributed to the low among‐population diversity we observed, since coding regions are generally more conserved and under greater selection pressure (Jehl et al., 2021; Makałowski & Boguski, 1998; Zhao et al., 2003); however, transcriptome data are commonly used to determine population structure of species like giant ragweed that lack a reference genome sequence or those with structurally complex genomes, and they provide added insights into transcript expression diversity (Hämälä et al., 2020; Okada et al., 2018; Ophir et al., 2014; Takahagi et al., 2016).

### 4.2 Multiple origins followed by local spread

Weedy populations derived from wild populations may originate from multiple, local independent origins or from a single origin with subsequent spreading of adapted biotypes across the landscape (Basu et al., 2004; Charbonneau et al., 2018; Ellstrand et al., 2010; Vigueira et al., 2013; Waselkov et al., 2020). Our data suggest that these pathways work simultaneously to contribute to the origins of weedy giant ragweed populations. Population structure and phylogenetic analyses both suggest that weedy populations from OH and IA‐MN originated independently, since weedy populations showed more genetic similarity with nearby wild populations than with distant weedy populations (Figure 1). The hypothesis that weediness can evolve independently across multiple regions is also supported by recent reports of independent origins for herbicide resistance across weedy giant ragweed populations (Van Horn et al., 2018) and the evolution of weedy sunflower biotypes (Kane & Rieseberg, 2008; Lai et al., 2008).

We can also infer widespread dispersal among weedy populations, based on clusters on the phylogeny including individuals from geographically distant weedy populations (e.g., OH4‐A and OH7‐A; IA4‐A, IA6‐A, and MN3‐A). Genetic similarity between populations was therefore relatively high, especially in the IA‐MN region. Thus, even if most weedy populations arose independently from nearby wild populations, some likely originated from more distant wild and/or weedy populations. Rapid spread of weedy giant ragweed populations in crop fields has been attributed in part to mechanical seed dispersal via harvesting equipment, particularly in regions with large farm sizes and where farmers outsource grain harvesting to third‐party operators who harvest from multiple farms (Chauvel et al., 2021; Vink et al., 2012).

Giant ragweed is monoecious, wind‐pollinated, and a producer of copious pollen, which may facilitate recurrent outcrossing between adjacent weedy and wild giant ragweed populations, (Bassett & Crompton, 1982), leading to gene flow and complicating the identification of population ancestry (Charbonneau et al., 2018; Kane & Rieseberg, 2008). Pollen‐mediated gene flow in giant ragweed has been observed at a rate of 3%–4% at 50 m from the pollen source (Ganie & Jhala, 2017) and over 30% at distances less than 0.76 m (Brabham et al., 2011; Ganie & Jhala, 2017). These results, and our own findings, suggest weedy giant ragweed populations can originate independently from wild populations, and once established, may then spread across fields and/or pass on weediness genes to wild populations through outcrossing.

### 4.3 Evidence for convergent evolution and the role of gene expression regulation in weed evolution

#### 4.3.1 Gene expression diversity

Gene expression regulation has been proposed to play a key role in the evolution of weeds (Charbonneau et al., 2018; Josephs et al., 2021; Lai et al., 2008; Vigueira et al., 2013). We found greater GED overall in wild versus weedy giant ragweed populations, possibly reflecting its evolution in riparian habitats subjected to frequent disturbance (Bassett & Crompton, 1982; Waselkov et al., 2020). Such a pattern could also result if genetic bottlenecks occur as wild plants invade crop fields, reducing population phenotypic and genetic variance.

Despite higher overall GED in wild giant ragweed populations, we identified individual genes for which GED was higher in weedy populations than in their paired wild populations (and vice versa). The annotated pathways of these two groups of genes showed considerable overlap, suggesting that the evolution of weediness may involve increases *or* decreases in expression diversity at the level of individual genes within a pathway. Future efforts to determine how such variation in GED may contribute to fitness differences at the population level would be worthwhile.

#### 4.3.2 Differential gene expression

Using the conventional DESeq2 approach, we identified relatively few DEGs and thus relatively little differentiation in gene expression among weedy and wild populations. The differentiation we did identify was mostly population‐specific, supporting the hypothesis of multiple, independent origins of weedy populations through different genetic means. These results are consistent with genomic and gene expression studies of weedy and wild sunflower populations in the U.S. (Bock et al., 2020; Drummond, 2018; Kane & Rieseberg, 2008; Lai et al., 2008), indicating that this pattern is not unique to our study system.

A shortcoming of the conventional DESeq2 approach in our study was using one wild giant ragweed transcriptome as the reference for mapping transcriptome reads from all other samples; this resulted in loss of transcripts that may be uniquely present or expressed in other giant ragweed weedy and wild transcriptomes. We rectified this issue by implementing a novel pipeline to retrieve and analyze both weedy and wild unmapped reads, identifying thousands of transcripts common to all our weedy populations and providing evidence for potential convergence among weedy populations and divergence from wild populations. Weedy traits in independently evolved weedy populations may thus arise through two sets of evolutionary mechanisms, one reflecting convergent evolution in response to common selection pressures (Huang et al., 2017; Thurber et al., 2013; Vigueira et al., 2013), and the other reflecting population‐specific evolutionary change shaped by the original genetic background, genetic drift, random mutations, and location‐specific selection pressures.

Many DEGs common across our weedy giant ragweed populations were involved in functional pathways presumably important for adapting to agricultural fields, such as seed germination, rhythm responses, vegetative phase change, and nitrogen assimilation. Seed germination, rhythm responses, and vegetative phase change functional pathways directly relate to prolonged seedling emergence timing characteristic of weedy giant ragweed. Common garden studies have shown clear phenotypic differences between weedy and wild giant ragweed in the duration of seedling emergence and onset of flowering (Hartnett et al., 1987; Schutte et al., 2008, 2012; Sprague et al., 2004), presumably because prolonged seedling emergence avoids some mortality from early‐season weed management practices and earlier flowering may be adaptive in ensuring reproduction before crop harvest. Geographic variation in agriculturally adaptive traits has been reported for this and other native weeds (Bravo et al., 2017; Waselkov et al., 2020) and may reflect regional variation in agrestal selection histories. In sunflowers, downregulation of defense genes in resource‐rich environments such as crop fields may liberate resources that enable increased competitiveness and fitness (Mayrose et al., 2011). We note that the transcript differences we detected in our study were found in leaf tissues harvested from seedlings grown under identical greenhouse conditions and thus are constitutive changes and do not simply reflect responses to environmental conditions. Future studies investigating gene expression in weedy versus wild giant ragweed across multiple tissue types and under varying environmental conditions will provide additional insight into the genes we identified here, perhaps detecting additional candidate “weediness genes.”

#### 4.3.3 Altered gene co‐expression pattern

The rewiring of gene co‐expression networks in cultivated species compared with their wild relatives has recently been hypothesized (Fajardo & Quecini, 2021; Jones & Vandepoele, 2020). Analogously, altered gene expression patterns in the transition to becoming weedy could result in variable co‐expression networks. Our analyses revealed eight such variable gene modules between weedy and wild giant ragweed populations, two of which are associated with herbicide resistance. One of these variable modules is involved in branched‐chain amino acid biosynthesis, an essential biochemical pathway targeted by some herbicides (Marion et al., 2017; Vila‐Aiub et al., 2009), and the other functions in xenobiotic transport, which is connected to herbicide resistance via detoxification of herbicides and other harmful compounds (Gaines et al., 2020). These gene expression network differences may reflect large‐scale evolutionary responses to the strong selection pressures exerted by repeated herbicide use in agricultural fields.

### 4.4 Herbicide resistance genes

Herbicide resistance is often implicated as a major facilitator of the rapid expansion of weedy biotypes in agricultural fields across species (Baucom, 2019; Délye, Jasieniuk, & Le Corre, 2013; Harre et al., 2017; Heap, 2021; Vink et al., 2012). We hypothesized that herbicide resistance contributes to the increased survival of weedy giant ragweed in agricultural fields and tested whether there was a correlation between weediness in giant ragweed and increased expression of major herbicide‐target genes, either due to changes in gene regulation or increased gene copy number (Gaines et al., 2010). Based on the similar expression levels of seven major herbicide‐target genes across weedy and wild populations, constitutive overexpression of these genes is not a significant contributor to weediness in giant ragweed (see also Moretti et al., 2018). Our study investigated the constitutive expression of these herbicide resistance genes under normal, untreated conditions, so additional studies are needed to determine whether these genes are overexpressed in weedy populations when exposed to the herbicides in question. We did find marginally significant overexpression of ALS gene transcripts in weedy compared with wild individuals (*p* = .052), suggesting constitutive overexpression of ALS may provide a survival advantage to weedy individuals in agriculture fields. Future genomic analyses of weedy and wild individuals will allow for the identification of genetic variation in regulatory regions contributing to this differential expression.

We also investigated SNP variation within herbicide resistance gene transcript sequences between weedy and wild giant ragweed individuals, which could contribute to resistance phenotypes. However, our investigation of ALS transcript sequences suggested that point mutations previously confirmed to confer resistance to ALS‐inhibiting herbicides were not the only factors driving the evolution of weediness in our study populations, since they occurred in both weedy and wild populations. Of course, the presence of herbicide‐resistant genotypes in wild populations could reflect the transmission of point mutations from resistant weedy individuals through pollen flow, but standing variation in wild giant ragweed populations that remains in the genome neutrally or is selected for directly via herbicide application to field margins and rights‐of‐way could also contribute (Drummond, 2018; Preston & Powles, 2002). Patzoldt and Tranel (2002) identified at least 15 different ALS alleles in a weedy giant ragweed population resistant to ALS inhibitors after only 3 years of herbicide selection pressure, and a high frequency of alleles conferred herbicide resistance (0.25). We do not know the herbicide application histories of our study populations, nor if our weedy population individuals were resistant to ALS‐inhibitor herbicides, but within‐population frequencies of ALS‐resistant genotypes frequencies were similarly high (up to 0.33; Table S10). Although this suggests past applications of ALS‐inhibitor herbicides may have influenced the evolution of even our wild populations, future herbicide treatment experiments, combined with genotyping analyses of ALS and other herbicide‐target genes in weedy and wild giant ragweed, are needed to clarify what role herbicide resistance plays in the evolution of weedy giant ragweed.

### 4.5 Weediness evolution in outcrossing native species

Recent surges in native, outcrossing species that have become weedy in North America indicate that some species are well positioned to become weedy or invasive in their native environment (Dekker, 2016; Leon et al., 2021; Waselkov et al., 2020). The diversity of adaptive traits such species exhibit, including resistance to multiple herbicides, nontarget site resistance, and morphological/phenological shifts, suggests these transitions often reflect complex genetic changes that may involve multiple genes, epistatic interactions, gene expression plasticity, and/or pleiotropy (Drummond, 2018; Hämälä et al., 2020; Leon et al., 2021; McGoey & Stinchcombe, 2021). The combination of large population sizes and substantial population genetic diversity in these species, including giant ragweed, likely enhances this genetic complexity and contributes to their transformation into problematic weeds. Intensive agriculture in North America is a relatively recent phenomenon, imposing strong and homogenous selection pressures over enormous geographic scales. This may help explain why native outcrossing species have emerged only recently as major agricultural weeds in North America and suggests that we can expect continued evolution of new weedy species in the future.

## AUTHOR CONTRIBUTIONS

**Bo Li:** Conceptualization (equal); data curation (lead); formal analysis (lead); investigation (equal); methodology (equal); validation (equal); visualization (lead); writing – original draft (lead). **Andrea R. Gschwend:** Conceptualization (equal); funding acquisition (supporting); investigation (equal); methodology (supporting); project administration (equal); supervision (equal); writing – review and editing (equal). **Stephen M. Hovick:** Conceptualization (equal); funding acquisition (equal); investigation (equal); methodology (equal); project administration (equal); supervision (equal); writing – review and editing (equal). **Amanda Gutek:** Data curation (equal); formal analysis (supporting); investigation (supporting). **Leah McHale:** Conceptualization (supporting); supervision (supporting); writing – review and editing (supporting). **S. Kent Harrison:** Conceptualization (supporting); funding acquisition (supporting); writing – review and editing (supporting). **Emilie E. Regnier:** Conceptualization (equal); funding acquisition (equal); investigation (equal); methodology (equal); project administration (equal); supervision (equal); writing – review and editing (equal).

## Supporting information


Figures S1‐S11

Table S1

Table S7

Supplementary Material

## Data Availability

The raw data used in this study have been deposited on NCBI under the BioProject (PRJNA726544).
