# Supplementary material for: Evolution of weedy giant ragweed (Ambrosia trifida): Multiple origins and gene expression variability facilitates weediness
Source: Ecol Evol. 2022 Dec 8;12(12):e9590. doi: 10.1002/ece3.9590 (PMC9731915; doi:10.1002/ece3.9590)
Supplement: Supplementary file 1 — Figures S1‐S11 [file ECE3-12-e9590-s003.pdf]

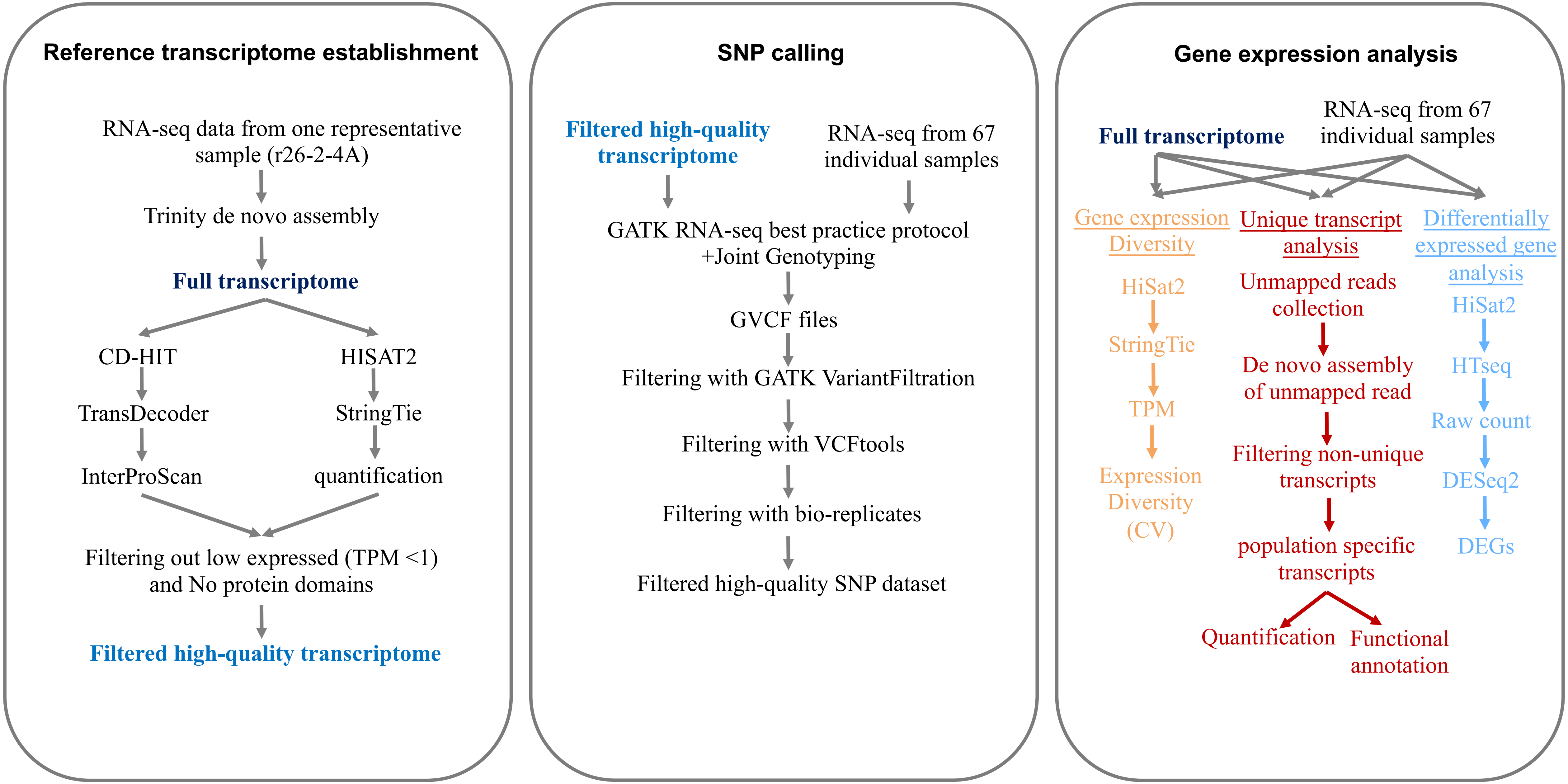

Supplemental Figure S1

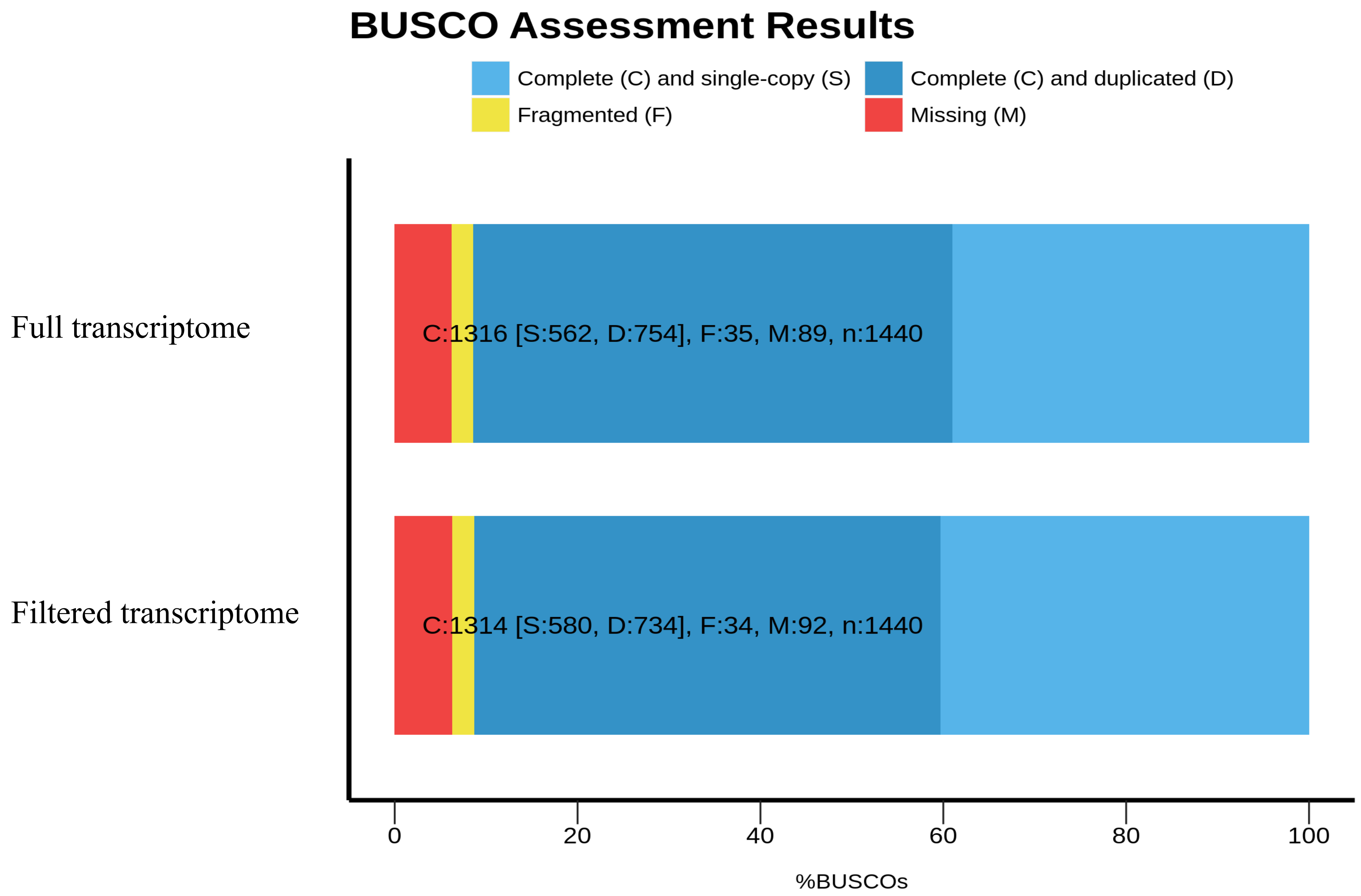

Supplemental Figure S2

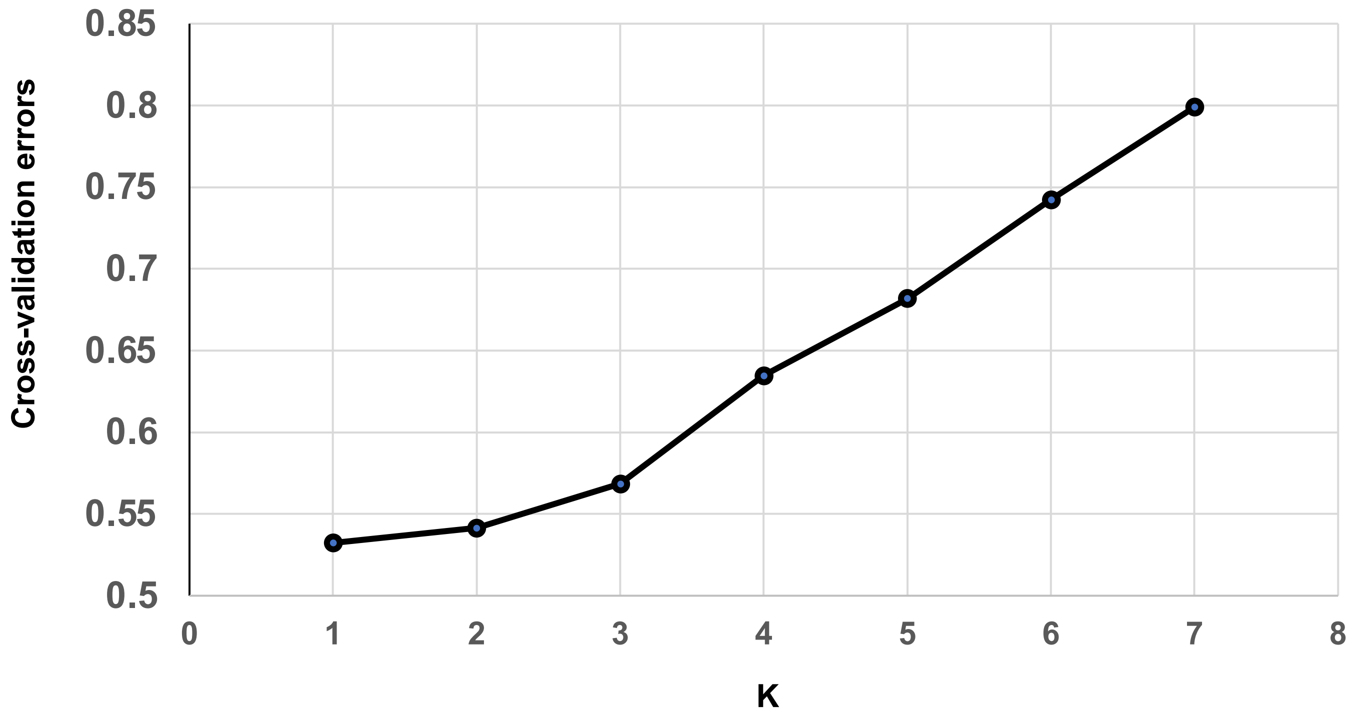

Supplemental Figure S3

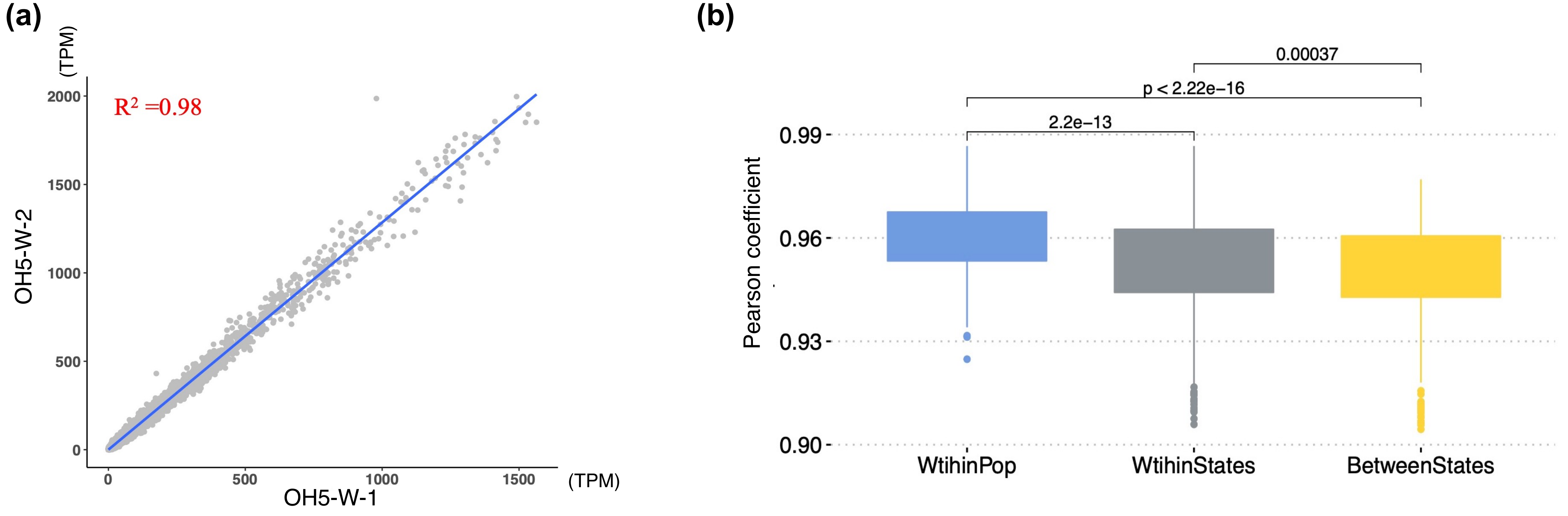

Supplemental Figure S4

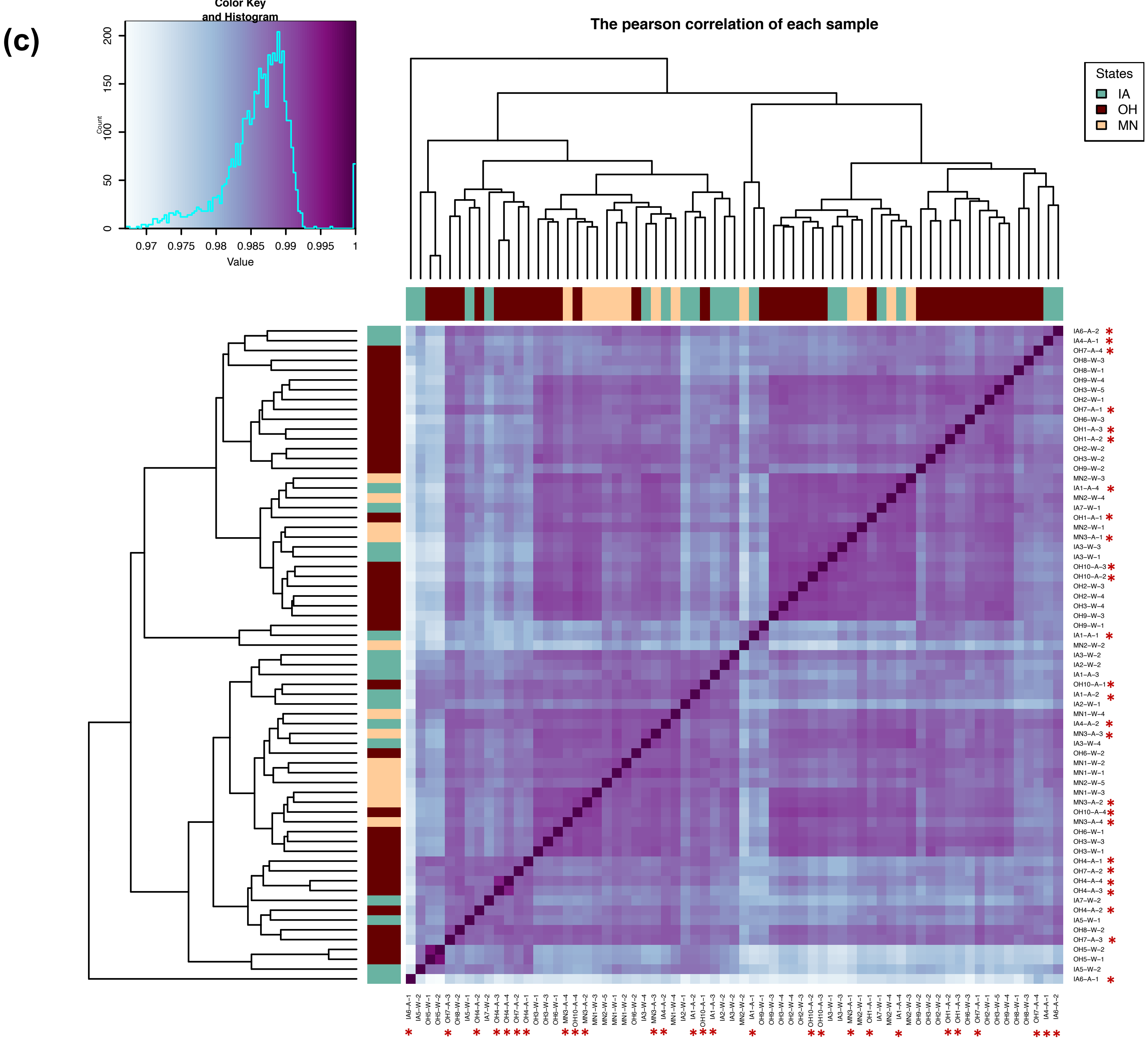

Supplemental Figure S4

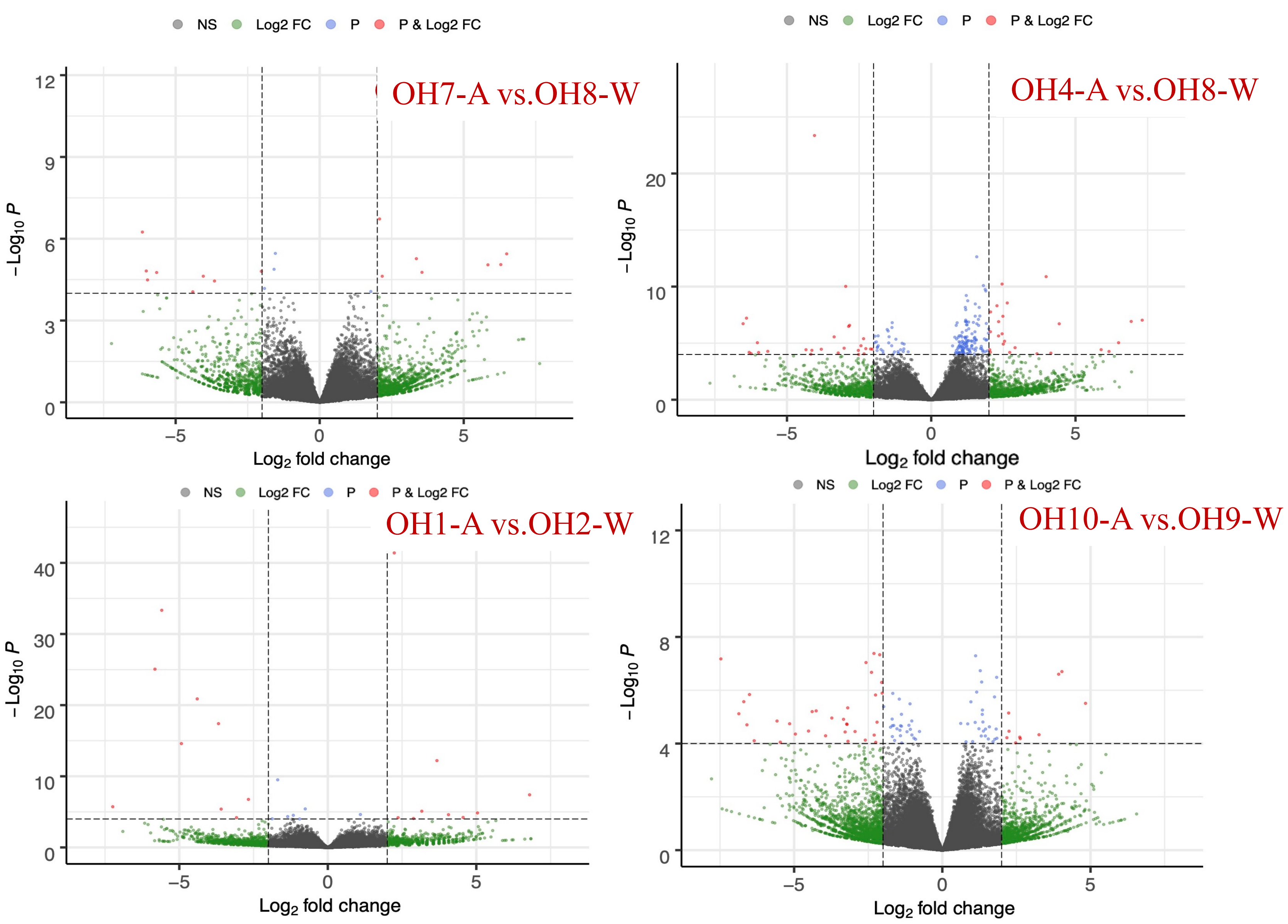

Supplemental Figure S5

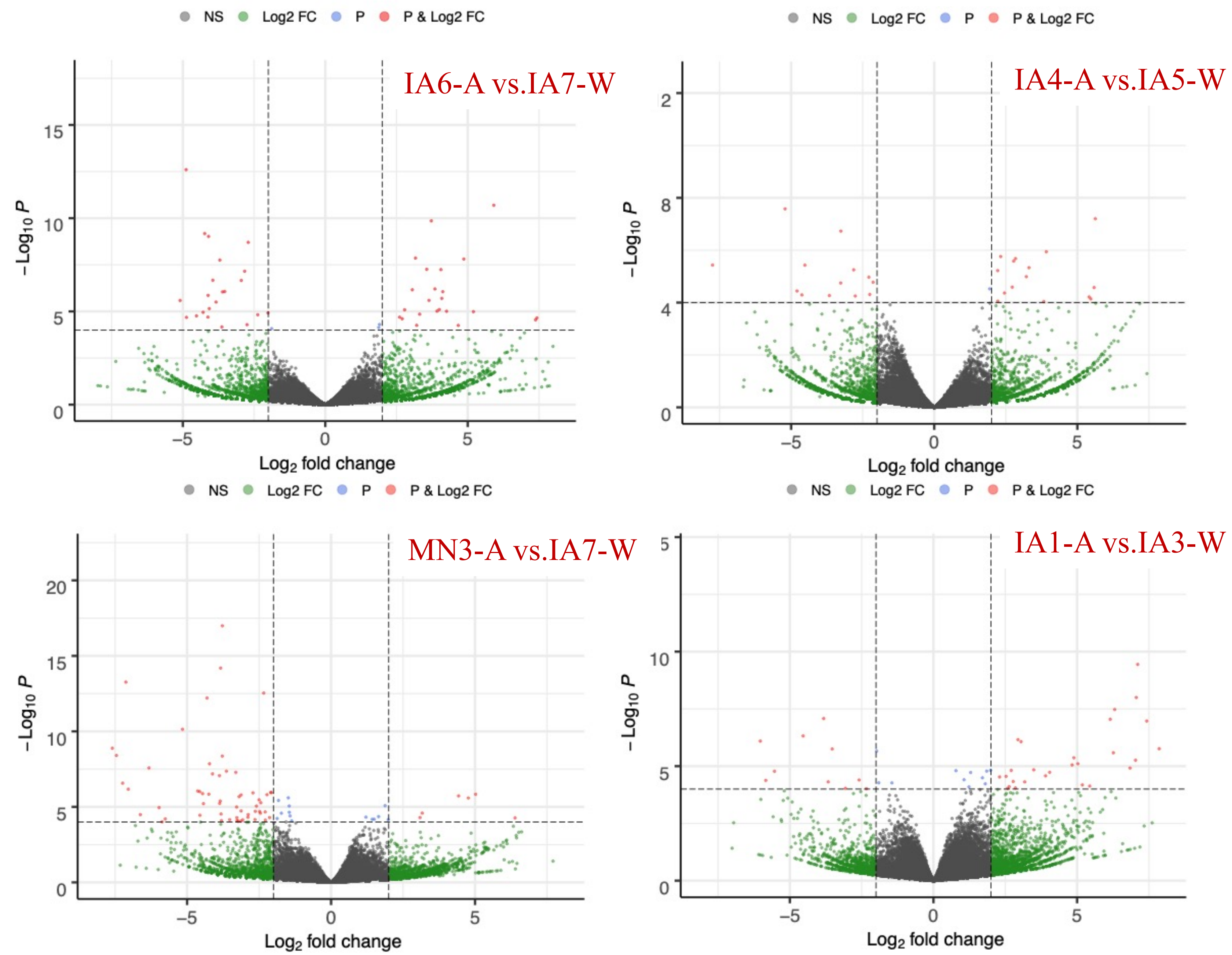

Supplemental Figure S6

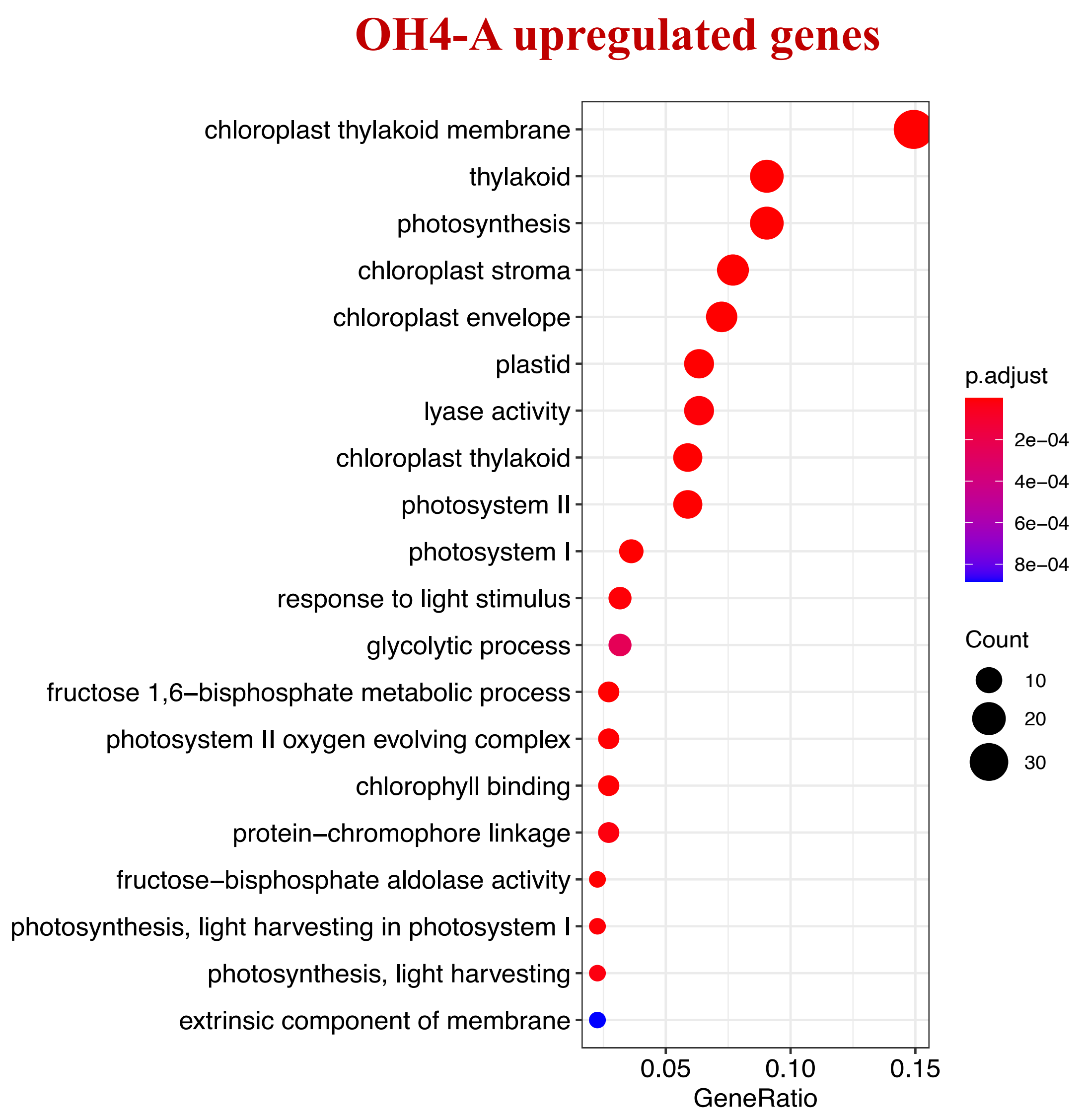

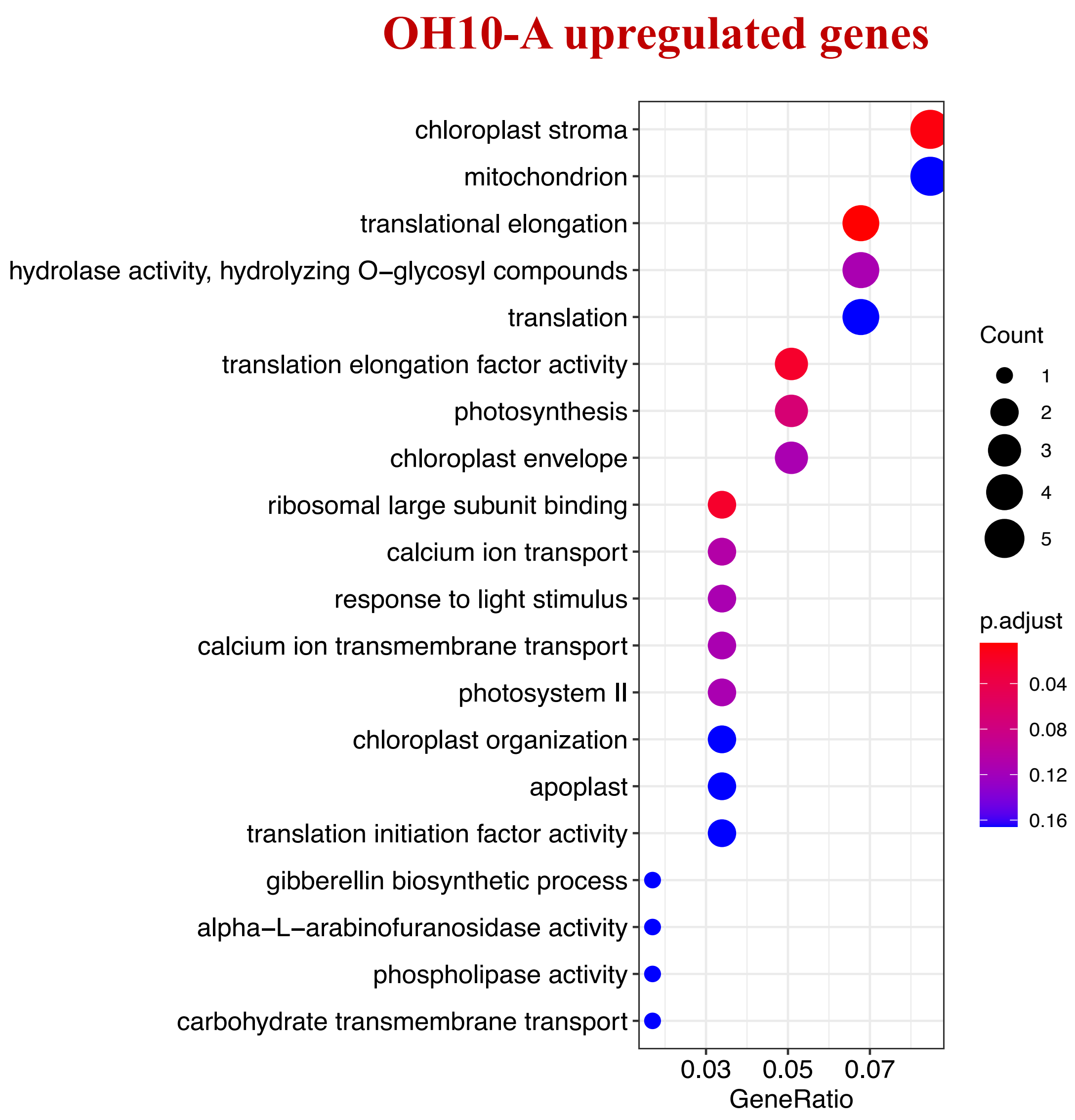

Supplemental Figure S7 a,b

IA4-A upregulated genes

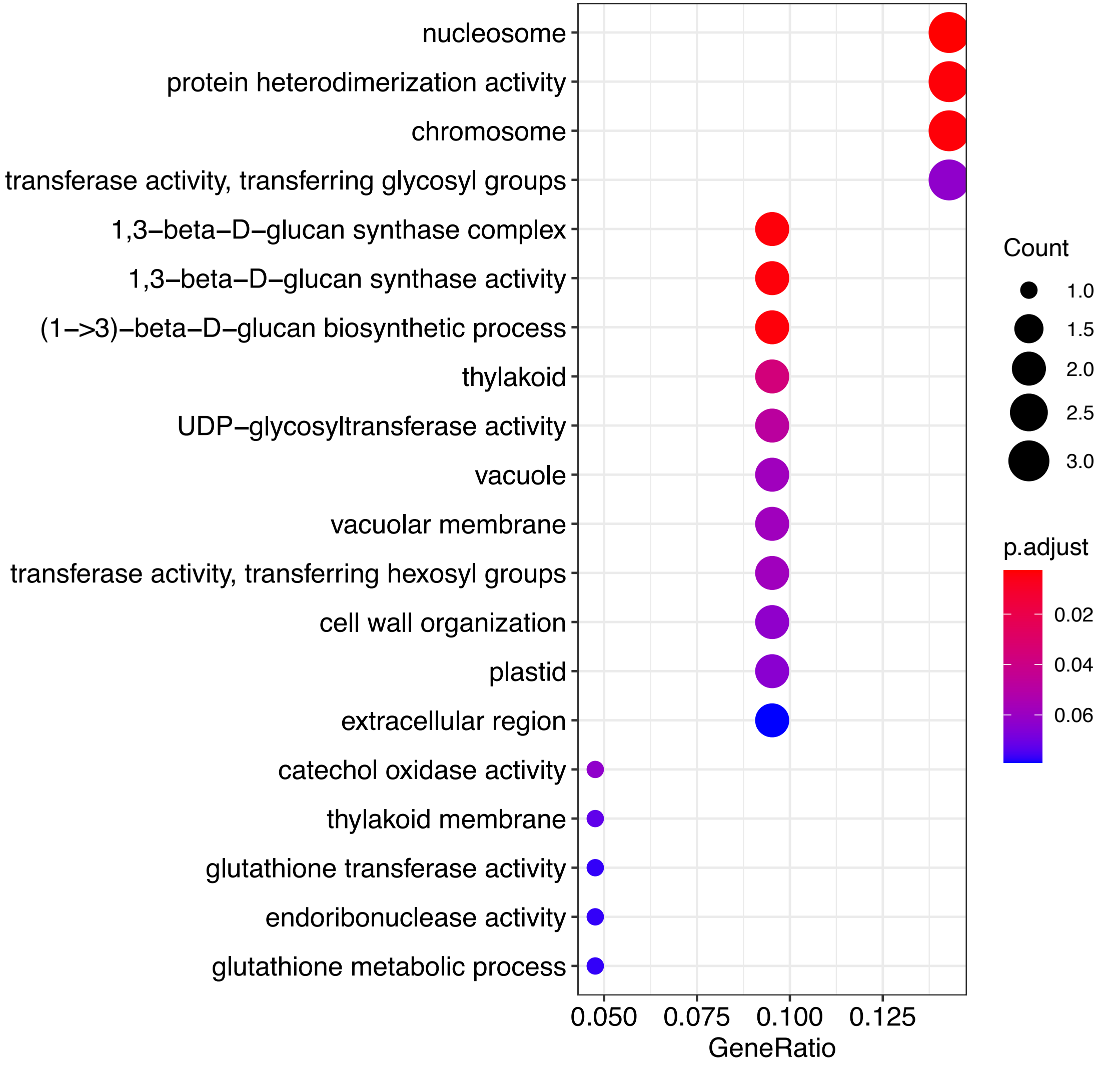

IA6-A upregulated genes

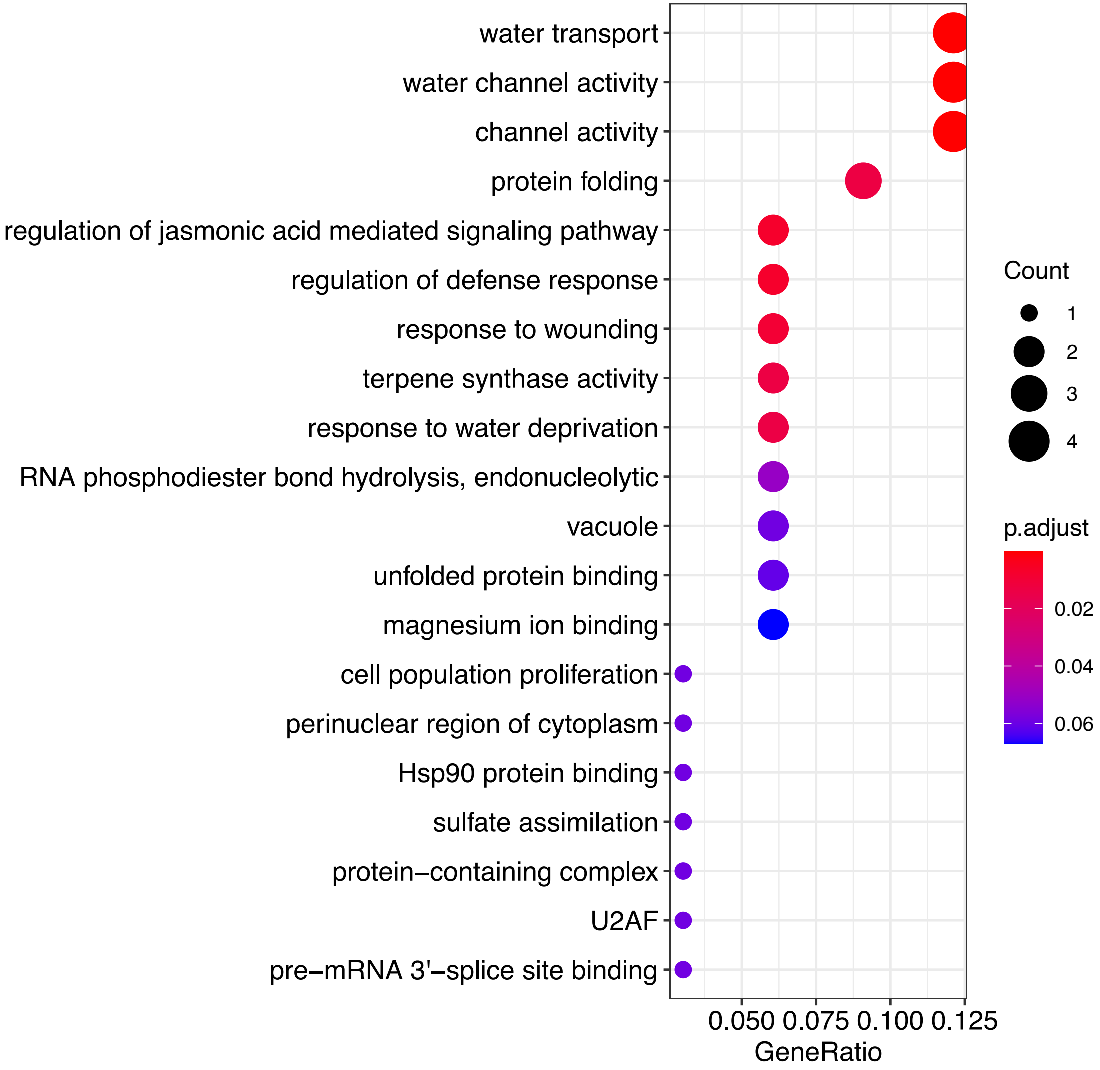

Supplemental Figure S7 c,d

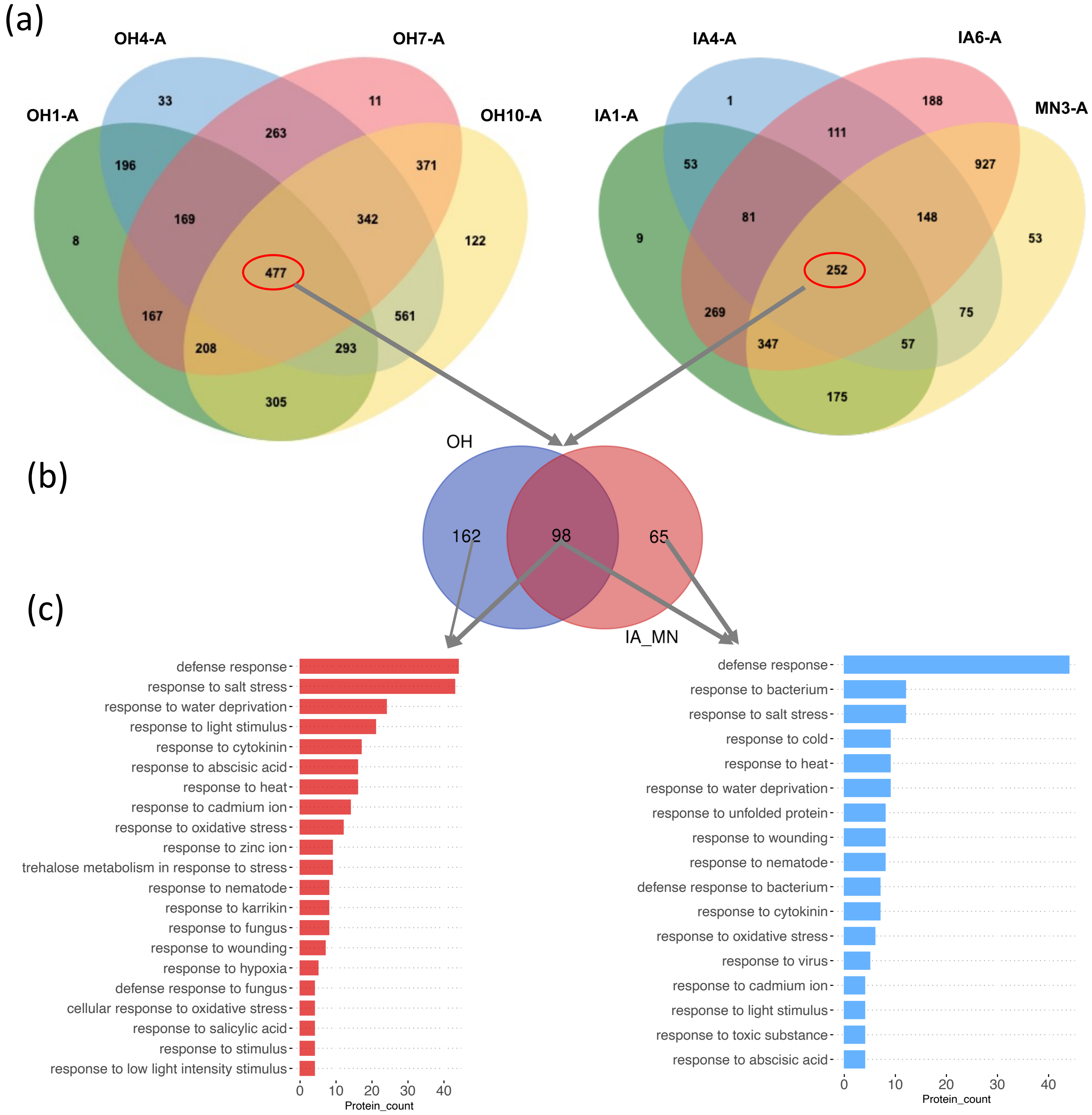

Supplemental Figure S8

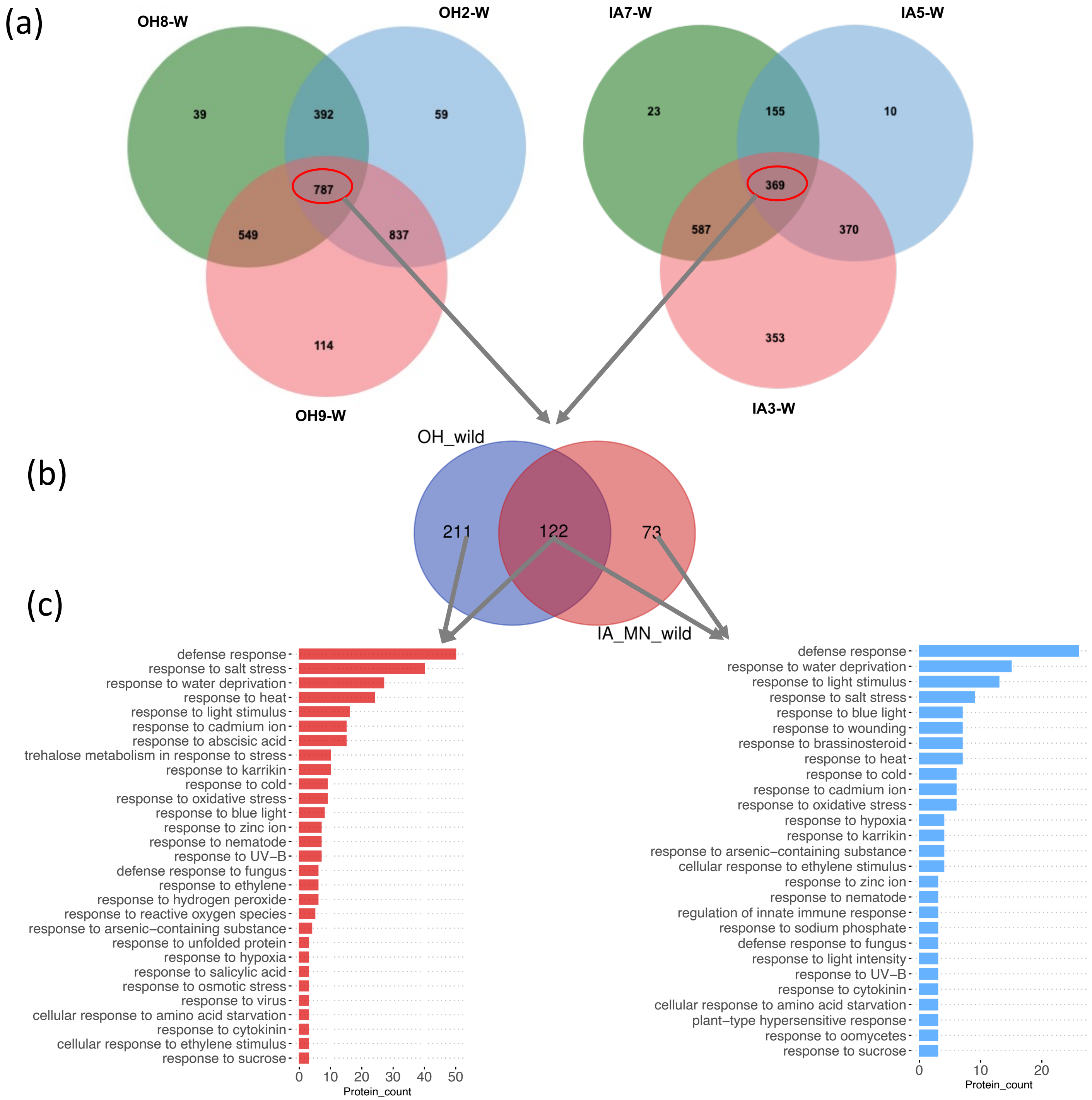

Supplemental Figure S9

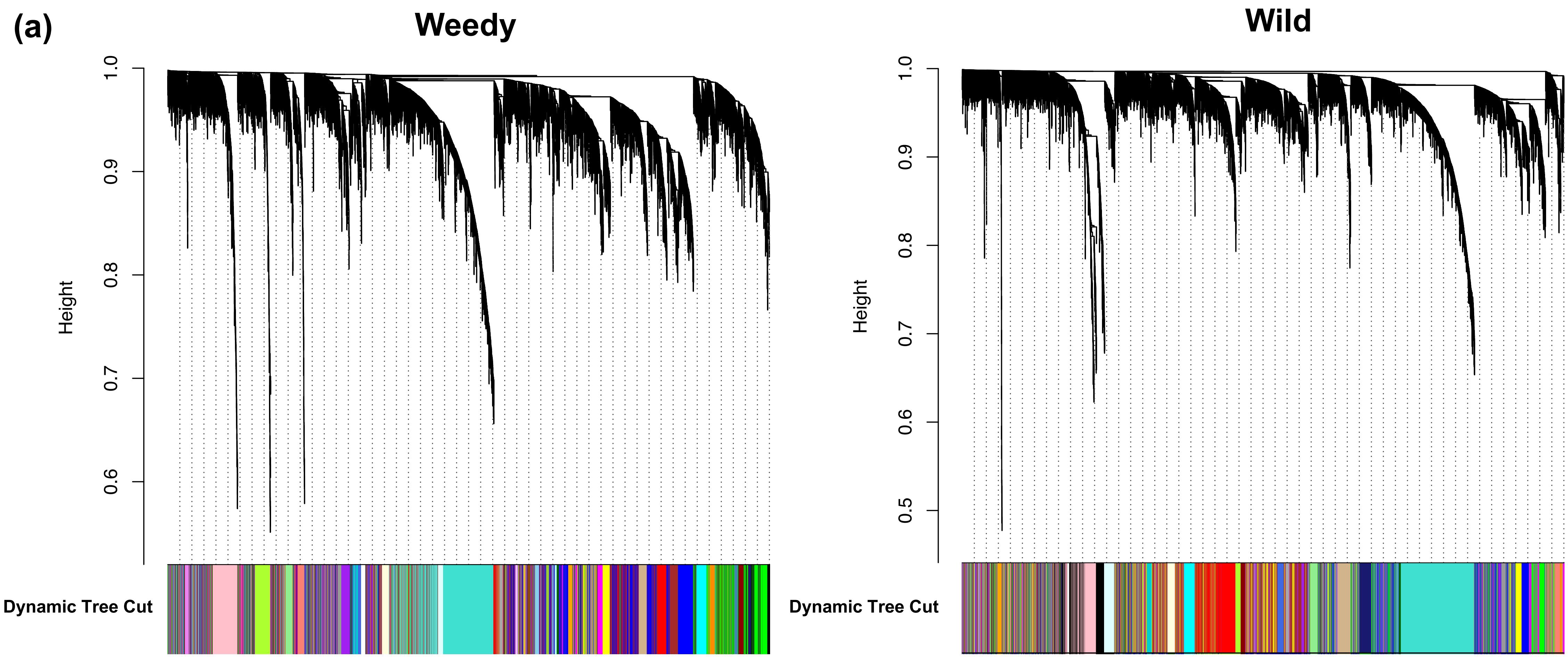

Supplemental Figure S10

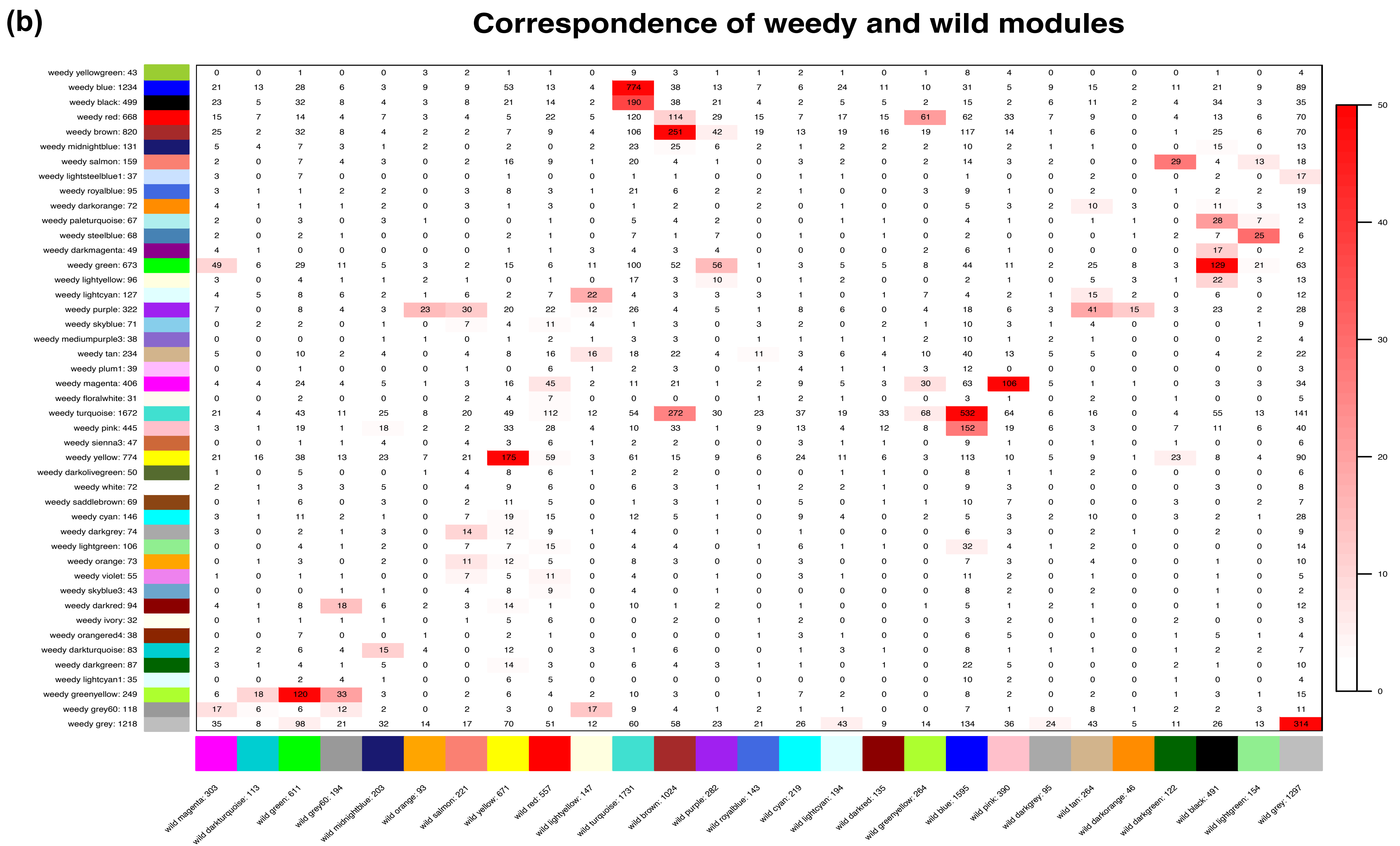

Supplemental Figure S10

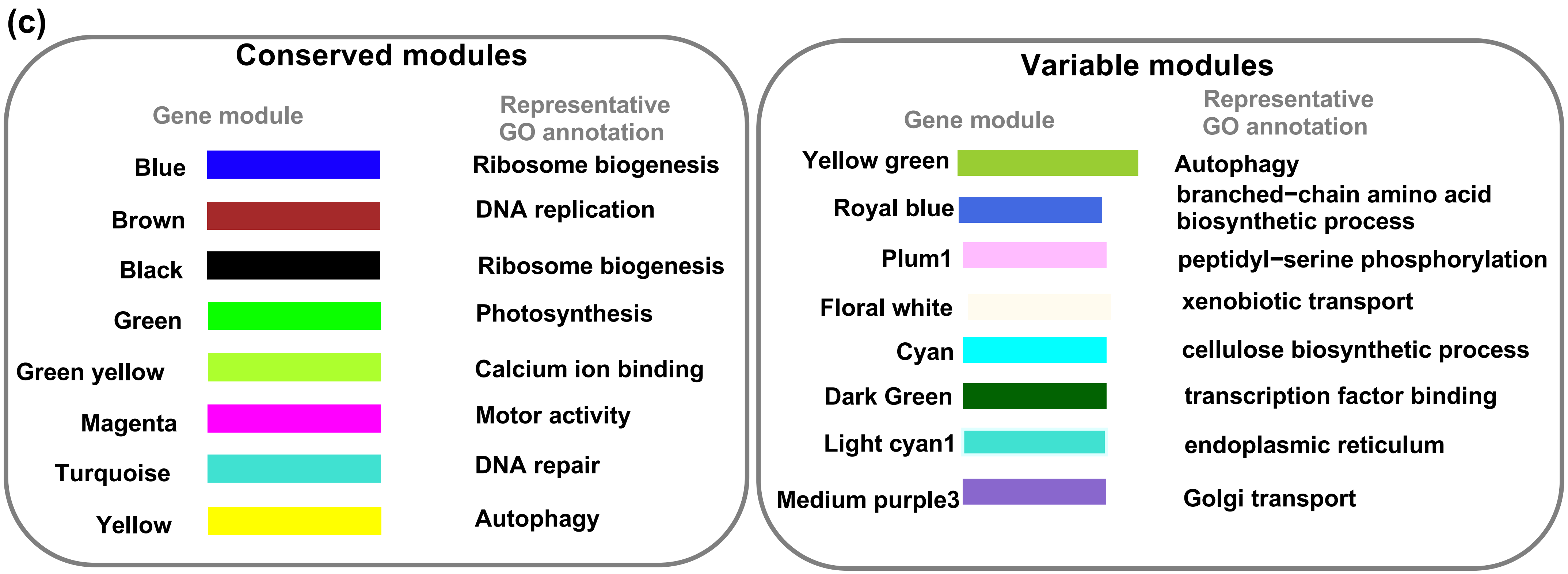

Supplemental Figure S10

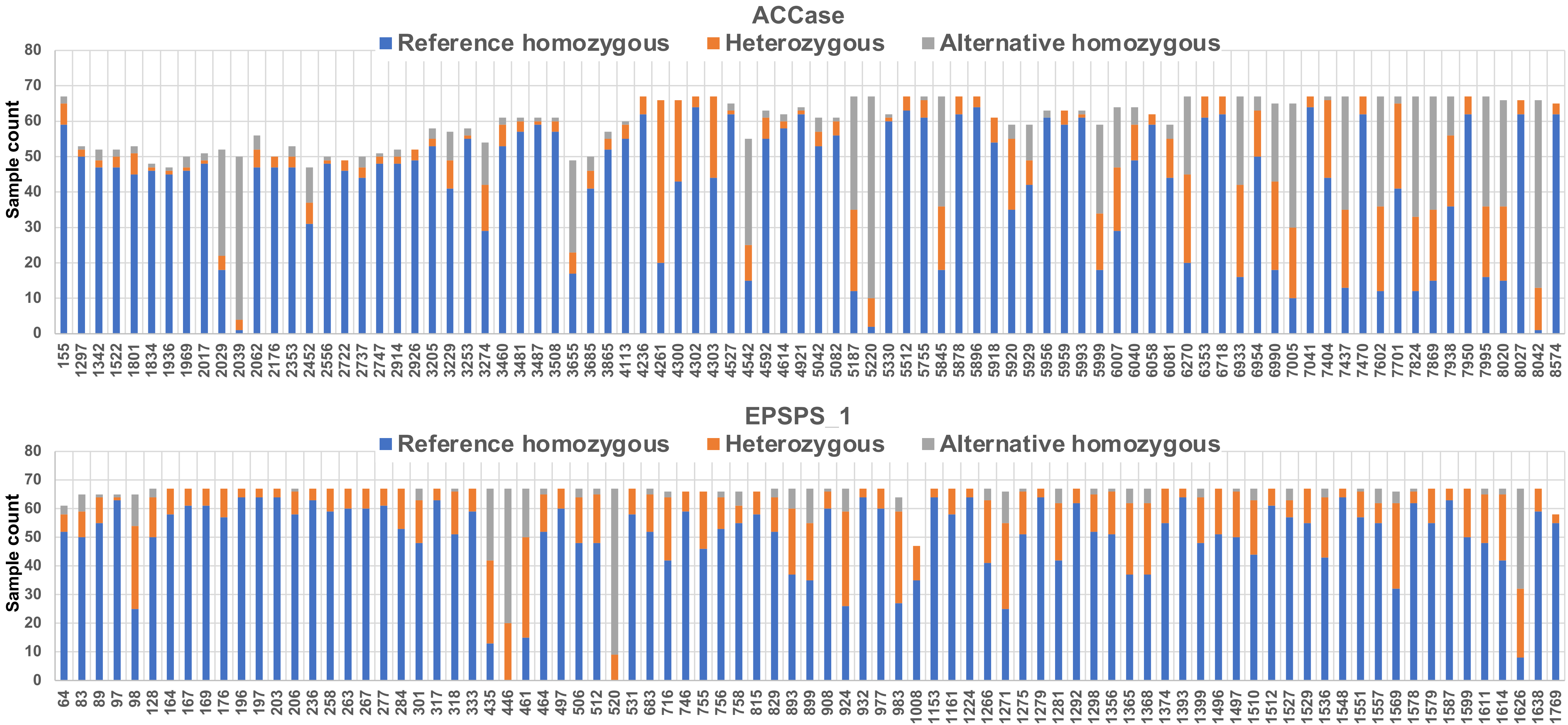

Supplemental Figure S11

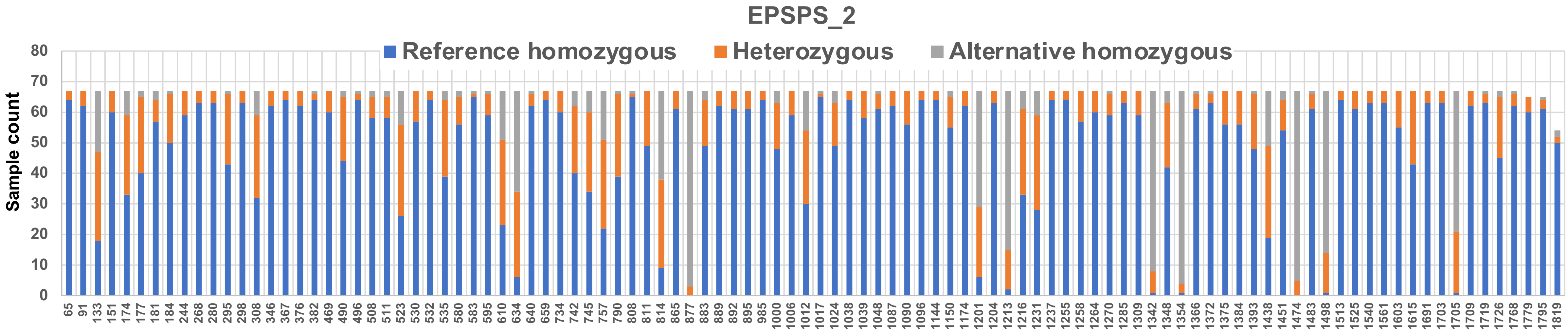

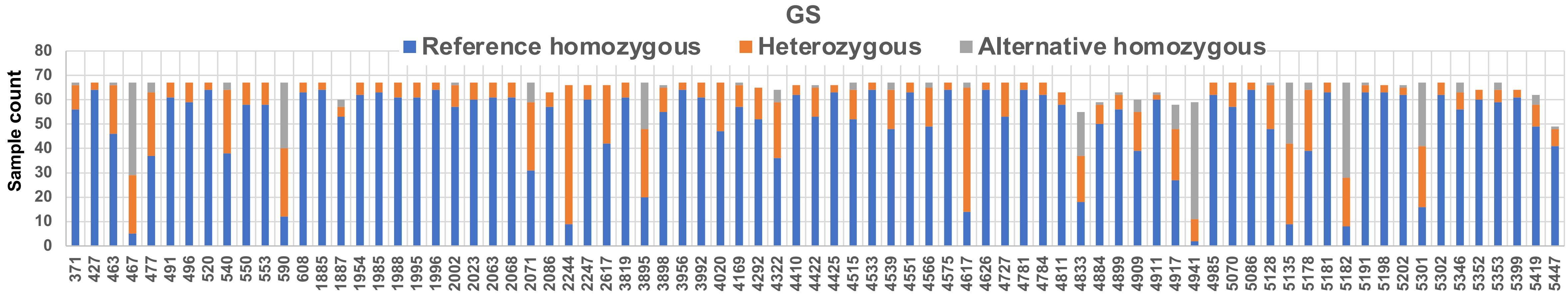

Supplemental Figure S11

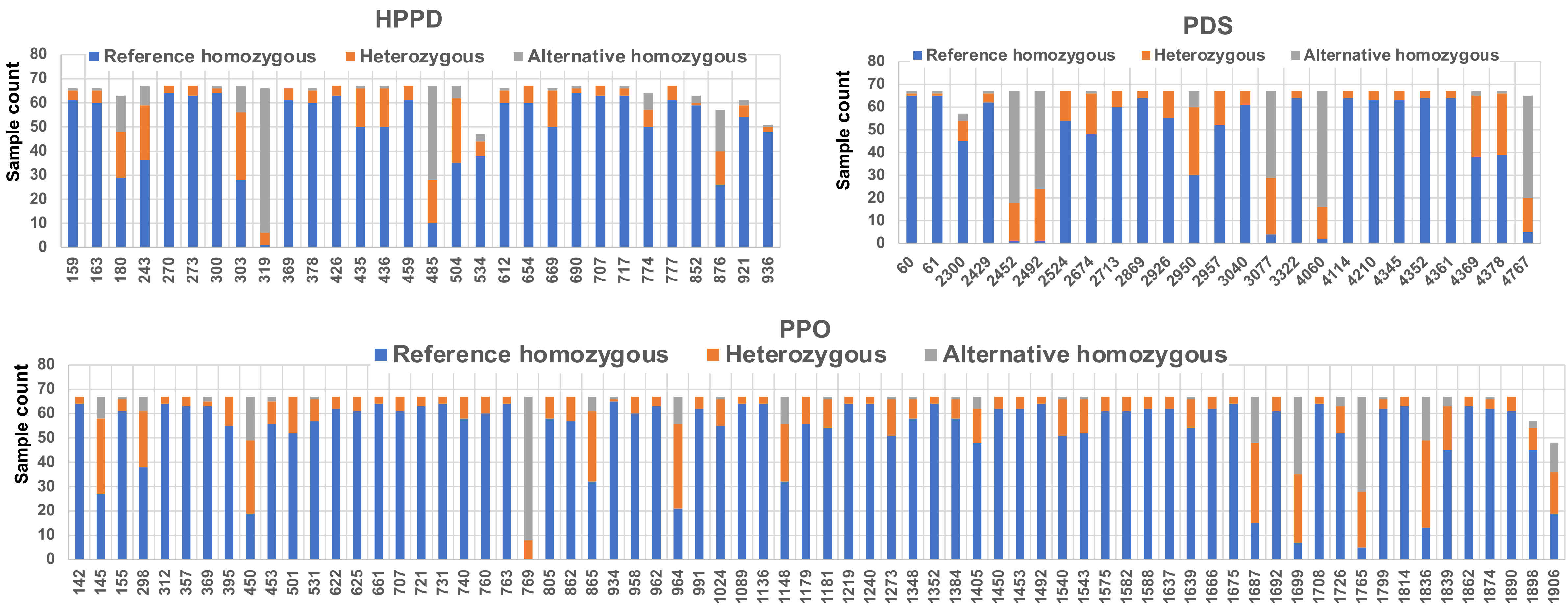

Supplemental Figure S11
