# Supplementary material for: Evolution of weedy giant ragweed (Ambrosia trifida): Multiple origins and gene expression variability facilitates weediness
Source: Ecol Evol. 2022 Dec 8;12(12):e9590. doi: 10.1002/ece3.9590 (PMC9731915; doi:10.1002/ece3.9590)
Supplement: Supplementary file 2 — Table S1 [file ECE3-12-e9590-s002.pdf]

Table S1. Population information of giant ragweed for RNA-seq.

Sample Number	Sample name	Population name	State	Habitats	Original Sample Name (NCBI submitted)	Note
**1**	OH1-A-1	OH1-A	Ohio	Weedy	Loc6-1-10A	
**2**	OH1-A-2	OH1-A	Ohio	Weedy	Loc6-2-8A	
**3**	OH1-A-3	OH1-A	Ohio	Weedy	Loc6-4-8A	
**4**	OH2-W-1	OH2-W	Ohio	Wild	r23-1-2B	
**5**	OH2-W-2	OH2-W	Ohio	Wild	r23-2-10B	
**6**	OH2-W-3	OH2-W	Ohio	Wild	r23-3-8A	
**7**	OH2-W-4	OH2-W	Ohio	Wild	r23-4-9B	
**8**	OH3-W-1	OH3-W	Ohio	Wild	r24-1-1A	
**9**	OH3-W-2	OH3-W	Ohio	Wild	r24-1-4B	
**10**	OH3-W-3	OH3-W	Ohio	Wild	r24-2-2A	
**11**	OH3-W-4	OH3-W	Ohio	Wild	r24-3-5B	
**12**	OH3-W-5	OH3-W	Ohio	Wild	r24-4.1A	
**13**	OH4-A-1	OH4-A	Ohio	Weedy	IPO-1-4B	OH4-A adjacent to OH5-W
**14**	OH4-A-2	OH4-A	Ohio	Weedy	IPO-2-3B	
**15**	OH4-A-3	OH4-A	Ohio	Weedy	IPO-3-5A	
**16**	OH4-A-4	OH4-A	Ohio	Weedy	IPO-3-5B	
**17**	OH5-W-1	OH5-W	Ohio	Wild	RPO-3-3A	OH5-W adjacent to OH4-A
**18**	OH5-W-2	OH5-W	Ohio	Wild	RPO-3-3B	
**19**	OH6-W-1	OH6-W	Ohio	Wild	r26-3-9A	
**20**	OH6-W-2	OH6-W	Ohio	Wild	r26-1-8B	
**21**	OH6-W-3	OH6-W	Ohio	Wild	r26-2-4A	Used for reference assembly
**22**	OH7-A-1	OH7-A	Ohio	Weedy	CIPO-2-1A	OH7-A adjacent to OH8-W
**23**	OH7-A-2	OH7-A	Ohio	Weedy	CIPO-3-3A	
**24**	OH7-A-3	OH7-A	Ohio	Weedy	CIPO-4-3A	
**25**	OH7-A-4	OH7-A	Ohio	Weedy	CIPO-4-2B	
**26**	OH8-W-1	OH8-W	Ohio	Wild	CUPO-2-5A	OH8-W adjacent to OH7-A
**27**	OH8-W-2	OH8-W	Ohio	Wild	CUPO-3-1B	
**28**	OH8-W-3	OH8-W	Ohio	Wild	CUPO-4-3A	
**29**	OH9-W-1	OH9-W	Ohio	Wild	rw2-1-10A	
**30**	OH9-W-2	OH9-W	Ohio	Wild	rw2-3-2A	
**31**	OH9-W-3	OH9-W	Ohio	Wild	rw2-2-7B	
**32**	OH9-W-4	OH9-W	Ohio	Wild	rw2-4-4A	
**33**	OH10-A-1	OH10-A	Ohio	Weedy	rw3-3-5A	
**34**	OH10-A-2	OH10-A	Ohio	Weedy	rw3-4-8B	
**35**	OH10-A-3	OH10-A	Ohio	Weedy	rw3-1-7A	

**36**	OH10-A-4	OH10-A	Ohio	Weedy	rw3-2-9A	
**37**	IA1-A-1	IA1-A	Iowa	Weedy	rw14-2-5B	
**38**	IA1-A-2	IA1-A	Iowa	Weedy	rw14-3-2A	
**39**	IA1-A-3	IA1-A	Iowa	Weedy	rw14-1-5B	
**40**	IA1-A-4	IA1-A	Iowa	Weedy	rw14-4-4B	
**41**	IA2-W-1	IA2-W	Iowa	Wild	rw15-2-5A	
**42**	IA2-W-2	IA2-W	Iowa	Wild	rw15-4-6A	
**43**	IA3-W-1	IA3-W	Iowa	Wild	rw16-1-5B	
**44**	IA3-W-2	IA3-W	Iowa	Wild	rw16-2-7A	
**45**	IA3-W-3	IA3-W	Iowa	Wild	rw16-3-5B	
**46**	IA3-W-4	IA3-W	Iowa	Wild	rw16-4-2B	
**47**	IA4-A-1	IA4-A	Iowa	Weedy	EII-1-3A	IA4-A adjacent to IA5-W
**58**	IA4-A-2	IA4-A	Iowa	Weedy	EII-4-5A	
**49**	IA5-W-1	IA5-W	Iowa	Wild	ERI-1-1A	IA5-W adjacent to IA4-A
**50**	IA5-W-2	IA5-W	Iowa	Wild	ERI-3-5A	
**51**	IA6-A-1	IA6-A	Iowa	Weedy	BII-1-3A	IA6-A adjacent to IA7-W
**52**	IA6-A-2	IA6-A	Iowa	Weedy	BII-4-5B	
**53**	IA7-W-1	IA7-W	Iowa	Wild	BUPI-1-1B	IA7-W adjacent to IA6-A
**54**	IA7-W-2	IA7-W	Iowa	Wild	BUPI-2-2A	
**55**	MN1-W-1	MN1-W	Minnesota	Wild	r21-1-8B	
**56**	MN1-W-2	MN1-W	Minnesota	Wild	r21-1-10A	
**57**	MN1-W-3	MN1-W	Minnesota	Wild	r21-3-4A	
**58**	MN1-W-4	MN1-W	Minnesota	Wild	r21-4-6A	
**59**	MN2-W-1	MN2-W	Minnesota	Wild	rw20-1-10A	
**60**	MN2-W-2	MN2-W	Minnesota	Wild	rw20-2-1A	
**61**	MN2-W-3	MN2-W	Minnesota	Wild	rw20-2-2A	
**62**	MN2-W-4	MN2-W	Minnesota	Wild	rw20-3-5A	
**63**	MN2-W-5	MN2-W	Minnesota	Wild	rw20-4-8B	
**64**	MN3-A-1	MN3-A	Minnesota	Weedy	rw19-1-5A	
**65**	MN3-A-2	MN3-A	Minnesota	Weedy	rw19-2-2A	
**66**	MN3-A-3	MN3-A	Minnesota	Weedy	rw19-3-4A	
**67**	MN3-A-4	MN3-A	Minnesota	Weedy	rw19-4-5B	

Table S2. Summary of reference transcriptomes for giant ragweed.

	Full transcriptome	Filtered transcriptome
**Total trinity genes**	66747	41669
**Total trinity transcripts**	144256	91296
**Percent GC (%)**	40.18	40.73
**Contig N10 (bp)**	3443	3652
**Contig N20 (bp)**	2703	2904
**Contig N30 (bp)**	2240	2429
**Contig N40 (bp)**	1912	2087
**Contig N50 (bp)**	1631	1809
**Median contig length (bp)**	772	1075
**Average contig (bp)**	1066.25	1295.18
**Total assembled bases (bp)**	153812893	118244712

Table S3. Summary of variant calling results.

**Sample Size**	**67**
GATK callset	3618989
SNPs	3267439
Indels	407949
Filter by vcftools	875381
Filter by biological replicates	616607
Ts/Tv	2.18

Table S4: Population statistics for each population.

Population	Latitude (N)	longitude(W)	N	Habitat	Ho	He	π
OH							
**OH1-A**	40.33	-83	3	Weedy	0.1138	0.1775	0.0024
**OH2-W**	40.11	-83.21	4	Wild	0.1275	0.1774	0.0024
**OH3-W**	40.09	-83.25	5	Wild	0.1261	0.1775	0.0026
**OH4-A**	39.84	-83.25	4	Weedy	0.1263	0.177	0.002
**OH5-W**	39.84	-83.25	2	Wild	0.0738	0.1743	NA
**OH6-W**	39.89	-83.32	3	Wild	0.1244	0.1775	0.0023
**OH7-A**	39.86	-83.66	4	Weedy	0.1317	0.1774	0.0027
**OH8-W**	39.86	-83.66	3	Wild	0.1244	0.1774	0.0027
**OH9-W**	40.5	-83.78	4	Wild	0.1363	0.1774	0.0024
**OH10-A**	40.82	-84.27	4	Weedy	0.1636	0.1779	0.0026
IA							
**IA1-A**	42.05	-90.35	4	Weedy	0.1617	0.1778	0.0025
**IA2-W**	42.12	-90.77	2	Wild	0.1816	0.1781	0.0027
**IA3-W**	42.18	-90.79	4	Wild	0.1584	0.1779	0.0025
**IA4-A**	42.66	-91.49	2	Weedy	0.1007	0.1761	0.002
**IA5-W**	42.66	-91.49	2	Wild	0.1174	0.1765	0.0022
**IA6-A**	41.54	-93.33	2	Weedy	0.1287	0.1771	0.0023
**IA7-W**	41.54	-93.33	2	Wild	0.1222	0.1769	0.0023
MN							
**MN1-W**	43.84	-92.3	4	Wild	0.125	0.1772	0.0024
**MN2-W**	44.02	-92.39	5	Wild	0.1252	0.1771	0.0022
**MN3-A**	44.01	-92.41	4	Weedy	0.1248	0.1773	0.0024

Table S5: Pairwise F_{ST} among giant ragweed populations

	IA1-A	IA2-W	IA3-W	IA4-A	IA5-W	IA6-A	IA7-W	MN1-W	MN2-W	MN3-A	OH1-A	OH10-A	OH2-W	OH3-W	OH4-A	OH6-W	OH7-A	OH8-W	OH9-W
IA1-A	-	0.045	0.063	0.067	0.068	0.063	0.059	0.059	0.052	0.04	0.087	0.061	0.07	0.08	0.109	0.071	0.072	0.091	0.058
IA2-W	0.045	-	0.032	0.038	0.047	0.04	0.039	0.03	0.014	0.011	0.059	0.043	0.044	0.058	0.095	0.063	0.061	0.07	0.034
IA3-W	0.063	0.032	-	0.054	0.057	0.05	0.049	0.048	0.039	0.033	0.077	0.052	0.061	0.07	0.1	0.064	0.072	0.083	0.049
IA4-A	0.067	0.038	0.054	-	0.023	0.002	0.011	0.033	0.02	0.004	0.054	0.055	0.041	0.059	0.1	0.055	0.059	0.065	0.042
IA5-W	0.068	0.047	0.057	0.023	-	0.022	0.008	0.032	0.029	0.006	0.042	0.048	0.04	0.049	0.086	0.05	0.049	0.058	0.032
IA6-A	0.063	0.04	0.05	0.002	0.022	-	0.005	0.032	0.024	0.002	0.048	0.046	0.036	0.051	0.089	0.054	0.048	0.054	0.037
IA7-W	0.059	0.039	0.049	0.011	0.008	0.005	-	0.022	0.023	0.003	0.041	0.043	0.037	0.05	0.085	0.047	0.047	0.054	0.029
MN1-W	0.059	0.03	0.048	0.033	0.032	0.032	0.022	-	0.03	0.017	0.066	0.05	0.051	0.063	0.089	0.045	0.064	0.072	0.041
MN2-W	0.052	0.014	0.039	0.02	0.029	0.024	0.023	0.03	-	0.016	0.061	0.044	0.044	0.058	0.084	0.038	0.059	0.067	0.036
MN3-A	0.04	0.011	0.033	0.004	0.006	0.002	-0.003	0.017	0.016	-	0.045	0.03	0.033	0.045	0.073	0.024	0.043	0.051	0.025
OH1-A	0.087	0.059	0.077	0.054	0.042	0.048	0.041	0.066	0.061	0.045	-	0.041	0.026	0.034	0.063	0.025	0.034	0.039	0.024
OH10-A	0.061	0.043	0.052	0.055	0.048	0.046	0.043	0.05	0.044	0.03	0.041	-	0.026	0.033	0.067	0.024	0.036	0.045	0.018
OH2-W	0.07	0.044	0.061	0.041	0.04	0.036	0.037	0.051	0.044	0.033	0.026	0.026	-	0.02	0.052	0.008	0.021	0.031	0.013
OH3-W	0.08	0.058	0.07	0.059	0.049	0.051	0.05	0.063	0.058	0.045	0.034	0.033	0.02	-	0.057	0.015	0.028	0.038	0.019
OH4-A	0.109	0.095	0.1	0.1	0.086	0.089	0.085	0.089	0.084	0.073	0.063	0.067	0.052	0.057	-	0.05	0.059	0.07	0.05
OH6-W	0.071	0.063	0.064	0.055	0.05	0.054	0.047	0.045	0.038	0.024	0.025	0.024	0.008	0.015	0.05	-	0.019	0.034	0.007
OH7-A	0.072	0.061	0.072	0.059	0.049	0.048	0.047	0.064	0.059	0.043	0.034	0.036	0.021	0.028	0.059	0.019	-	0.023	0.022
OH8-W	0.091	0.07	0.083	0.065	0.058	0.054	0.054	0.072	0.067	0.051	0.039	0.045	0.031	0.038	0.07	0.034	0.023	-	0.03
OH9-W	0.058	0.034	0.049	0.042	0.032	0.037	0.029	0.041	0.036	0.025	0.024	0.018	0.013	0.019	0.05	0.007	0.022	0.03	-

Table S6. de novo assembly of unmapped reads to detect unique transcripts for paired weedy populations.

Population	Sample name	Mapping rate (%)	Total assembled transcript	Unique transcript	Unique transcript with ORF
**OH1-A**	**OH1-A-1**	79.71	43242	8537	2936
	**OH1-A-2**	78.98			
	**OH1-A-3**	78.91			
**OH4-A**	**OH4-A-1**	79.84	45131	9692	3313
	**OH4-A-2**	69.75			
	**OH4-A-3**	79.04			
	**OH4-A-4**	78.77			
**OH7-A**	**OH7-A-1**	79.29	49919	11457	4177
	**OH7-A-2**	79.2			
	**OH7-A-3**	80.09			
	**OH7-A-4**	77.13			
**OH10-A**	**OH10-A-1**	77.34	59710	23492	6155
	**OH10-A-2**	76.91			
	**OH10-A-3**	70.45			
	**OH10-A-4**	73.33			
**IA1-A**	**IA1-A-1**	67.19	61346	24639	6345
	**IA1-A-2**	66.98			
	**IA1-A-3**	64.57			
	**IA1-A-4**	69.87			
**IA4-A**	**IA4-A-1**	61.35	26694	4510	1136
	**IA4-A-2**	63.66			
**IA6-A**	**IA6-A-1**	64.81	34570	7404	1969
	**IA6-A-2**	65.65			
**MN3-A**	**MN3-A-1**	62.28	47764	13457	4090
	**MN3-A-2**	61.58			
	**MN3-A-3**	62.46			
	**MN3-A-4**	63.9			

Table_S7_Functional_annotation_summary (see an additional Excel spreadsheet).

Table S8. de novo assembly of unmapped reads to detect unique transcripts for paired wild populations.

**Population**	**Sample name**	**Mapping rate (%)**	**Total assembled transcript**	**Unique transcript**	**Unique transcript with ORF**
**OH2-W**	**OH2-W-1**	79.07	48398	13402	4200
	**OH2-W-2**	79.34			
	**OH2-W-3**	79.00			
	**OH2-W-4**	74.85			
**OH8-W**	**OH8-W-1**	79.99	53141	11327	3386
	**OH8-W-2**	79.43			
	**OH8-W-3**	79.42			
**OH9-W**	**OH9-W-1**	79.44	59290	21841	5653
	**OH9-W-2**	78.98			
	**OH9-W-3**	79.19			
	**OH9-W-4**	76.91			
**IA3-W**	**IA3-W-1**	70.94	59043	22891	6495
	**IA3-W-2**	62.87			
	**IA3-W-3**	68.56			
	**IA3-W-4**	66.38			
**IA5-W**	**IA5-W-1**	78.94	27795	6163	1524
	**IA5-W-2**	79.65			
**IA7-W**	**IA7-W-1**	79.80	32657	7491	2092
	**IA7-W-2**	78.87			

Table S9. Comparison of overlaps between functional pathways and unique transcripts.

	Shared unique transcripts of OH weedy populations	Shared unique transcripts of OH wild populations
Functional categories	260	333
Overlapped functional categories	160	160
Specific functional categories	100	173
**Overall overlap rate**	**54.0%**	
Total gene counts	2317	2640
Shared gene counts	614 (26.5%)	500 (18.9%)
Specific gene count	1703 (73.5%)	2140 (81.1%)
**Overall overlap rate**	**22.5%**	

Table S10. Herbicide resistance mutation genotypes of ALS gene in giant ragweed population.

POS[1]	304	349	765	766	765+766	1897	
REF[2]	G	C	C	C	CC	G	
ALT[3]	T	T	T	T	TT	T	
Substitutions[4]	**Arg-26-Leu**	**Ser-41-Phe**	**Pro-197-Ser**	**Pro-197-Leu**	**Pro-197-Phe**	**Trp-574-Leu**	
Samples	Genotype	Genotype	Genotype	Genotype	Genotype	Genotype	**Habitat**
OH1-A-1			TT				Weedy
OH1-A-3	GT					GT	Weedy
OH4-A-1	GT						Weedy
OH4-A-4		CT					Weedy
OH7-A-1		CT					Weedy
OH7-A-4						GT	Weedy
OH10-A-1	GT						Weedy
IA1-A-1					CT/CT		Weedy
IA1-A-3			CT				Weedy
IA1-A-4					CT/CT		Weedy
IA6-A-1		CT					Weedy
IA6-A-2		CT	CT				Weedy
OH2-W-1	TT				TT/TT		Wild
OH2-W-2	GT				CT/CT		Wild
OH2-W-3	TT				TT/TT		Wild
OH2-W-4						GT	Wild
OH3-W-3	GT					GT	Wild
OH3-W-4	GT						Wild
OH3-W-5		CT					Wild
OH6-W-1	GT					GT	Wild
OH6-W-2				CT			Wild
OH6-W-3	GT						Wild
IA5-W-1		CT					Wild
IA7-W-1			CT				Wild
MN2-W-5		CT					Wild

Note: for each sample, which have AA substitution, the mutation genotypes are listed. Reference homozygous genotypes are omitted. Green background highlights these mutations conferring resistance are experimentally confirmed in previous studies. 1. SNP position across ALS gene sequence. 2.Reference genotype. 3. Alternative genotype. 4. Amino acid alteration due to this SNP mutation.

Supplemental Table S11. Significant different genotype frequency between weedy and wild samples. Fisher's exact test is used to test if genotypes on each polymorphic site are significant different between weedy and wild populations.

Gene	Position	Ref SNP	Alt SNP	Ref homozygous genotype	Heterozygous genotype	Alt homozygous genotype	Unknown genotype	Weedy Ref Allele	Weedy Alt Allele	Wild Ref Allele	Wild Alt Allele	Fisher's exact test p-value	* 0.05 ** 0.01 *** 0.001
**ACCase**	155	T	C	59	6	2	0	**53**	**1**	**71**	**9**	**0.049**	*
	2353	A	G	47	3	3	14	**38**	**0**	**59**	**9**	**0.025**	*
	3205	G	A	53	2	3	9	**40**	**8**	**68**	**0**	**0.001**	***
	3229	G	T	41	8	8	10	**33**	**17**	**57**	**7**	**0.005**	**
	3460	G	A	53	6	2	6	**40**	**8**	**72**	**2**	**0.014**	*
	3481	A	G	57	3	1	6	**41**	**5**	**76**	**0**	**0.007**	**
	3685	A	G	41	5	4	17	**42**	**2**	**45**	**11**	**0.035**	*
	4592	C	A	55	6	2	4	**41**	**7**	**75**	**3**	**0.042**	*
**ALS**													
	204	A	G	46	5	0	16	**37**	**5**	**60**	**0**	**0.010**	*
	401	C	A	57	10	0	0	**53**	**1**	**71**	**9**	**0.049**	*
	557	T	C	15	23	29	0	**15**	**39**	**38**	**42**	**0.030**	*
	572	T	C	9	26	32	0	**24**	**30**	**20**	**60**	**0.024**	*
	587	A	G	61	6	0	0	**49**	**5**	**79**	**1**	**0.039**	*
	1064	T	A	62	4	1	0	**49**	**5**	**79**	**1**	**0.039**	*
	1532	G	A	59	7	1	0	**54**	**0**	**71**	**9**	**0.011**	*
	1591	A	G	60	7	0	0	**48**	**6**	**79**	**1**	**0.017**	*
	1973	C	T	44	19	4	0	**37**	**17**	**70**	**10**	**0.009**	**
**EPSPS_1**													
	318	A	G	51	15	1	0	**43**	**11**	**74**	**6**	**0.036**	*
	756	G	A	53	11	2	1	**50**	**2**	**67**	**13**	**0.046**	*
**EPSPS_2**													
	1090	A	G	56	11	0	0	**53**	**1**	**70**	**10**	**0.050**	*

	1150	C	T	55	10	2	0	**43**	**11**	**77**	**3**	**0.003**	**
	1201	T	C	6	23	38	0	**9**	**45**	**26**	**54**	**0.047**	*
	1819	A	G	50	2	2	13	**40**	**6**	**62**	**0**	**0.005**	**
**GS**													
	4169	A	G	57	9	1	0	**53**	**1**	**70**	**10**	**0.050**	*
**HPPD**													
	534	C	T	38	6	3	20	**35**	**1**	**47**	**11**	**0.026**	*
**PDS**													
	2300	C	T	45	9	3	10	**46**	**2**	**53**	**13**	**0.023**	*
	2492	C	A	1	23	43	0	**16**	**38**	**9**	**71**	**0.012**	*
	2926	G	A	55	12	0	0	**54**	**0**	**68**	**12**	**0.002**	**
**PPO**													
	155	C	T	61	5	1	0	**48**	**6**	**79**	**1**	**0.017**	*
	298	C	T	38	23	6	0	**45**	**9**	**54**	**26**	**0.047**	*
	805	T	C	58	9	0	0	**46**	**8**	**79**	**1**	**0.003**	**
	1405	T	G	48	14	5	0	**37**	**17**	**73**	**7**	**0.001**	**

Note:
Position: SNP position on transcripts
Ref SNP: nucleotide on reference transcripts
Alt SNP: nucleotide on sample reads
Ref homozygous genotype: sample count with homozygous reference genotype (0/0)
Heterozygous genotype: sample count with heterozygous genotype (0/1)
Alt homozygous genotype: sample count with homozygous alternative genotype (1/1)
Unknown genotype: missing data
Weedy Ref Allele: sample count in weedy population with reference alleles (0)
Weedy Alt Allele: sample count in weedy population with alternative alleles (1)
Wild Ref Allele: sample count in wild population with reference alleles (0)
Wild Alt Allele: sample count in wild population with alternative alleles (1)
