# Supplementary material for: Evolution of weedy giant ragweed (Ambrosia trifida): Multiple origins and gene expression variability facilitates weediness
Source: Ecol Evol. 2022 Dec 8;12(12):e9590. doi: 10.1002/ece3.9590 (PMC9731915; doi:10.1002/ece3.9590)
Supplement: Supplementary file 4 — Supplementary Material [file ECE3-12-e9590-s004.pdf]

Fig. S1 Research pipeline for transcriptome assembly, variants calling, and gene differential expression analysis. The full transcriptome was first established, and a high-quality filtered transcriptome was then constructed by removing poor quality transcripts without external evidence to support their retention. The filtered transcriptome was used in variant calling for improving SNP calling accuracy. The full transcriptome was used for gene expression analysis, according to the Trinity development team recommendations.

Fig. S2 BUSCO assessment on the full and filtered reference transcriptome of giant ragweed.

Fig. S3 Cross-validation errors calculated by ADMIXTURE. K=1 and K=2 were evaluated to be the best and second best assumptions for population structure of giant ragweed.

Fig. S4 RNA-seq data analysis. (a) Gene expression correlation between two biological replicate samples from the OH5-W population. (b) Correlation coefficient on expression for samples within populations, within states, and between states. Correlation coefficient is significantly higher within populations than among samples within states and higher than samples between states. This pattern agrees with the geographic distribution, where adjacent samples show higher similarity in expression pattern. (c) Dendrogram plot shows sample clustering according to correlation coefficient.

Fig. S5 Volcano plot for differentially expressed genes identified in four weedy-wild population pairs in the OH subgroup.

Fig. S6 Volcano plot for differentially expressed genes identified in four weedy-wild population pairs in the IA-MN subgroup.

Fig. S7 Functional (GO) enrichment analysis of upregulated genes in OH4-A, OH10-A, IA4-A and IA6-A populations. Gene ratio is the number of genes in the test group divided by those in the background. Count refers to the transcript number. P.adjust values account for FDR. Few enriched functional categories were detected for other weedy populations, thus the results from these populations are not shown here.

Fig. S8 Gene clustering analysis identified orthologous gene groups from common functional categories among weedy populations from OH and IA-MN. (a) Homolog detection with protein sequences from unique transcripts in each population identified 477 and 252 shared orthologous gene groups across the four weedy populations in OH and IA-MN, respectively. (b) Functional annotation on overlapped homologous groups. The results showed these genes can be assigned into 260 and 163 different GO functional groups, with 98 shared functional categories. (c) Highlighting the defense and biotic and abiotic resistance functions shared by OH and IA-MN weedy populations. Within the shared functional groups, responses to biotic and abiotic stressors were found to be overrepresented in weedy populations from both OH and IA-MN.

Fig. S9 Gene clustering analysis identified orthologous gene groups from common functional categories among wild populations from OH and IA-MN. (a) Homolog detection with protein sequences from unique transcripts in each population. 787 and 369 shared orthologous gene groups were found across the three wild populations (two weedy-wild pairs from each region

shared the same wild population) in OH and IA-MN, respectively. (b) Functional annotation on overlapped homologous groups. The results showed these genes can be assigned into 333 and 195 different GO functional groups, with 122 shared functional categories. (c) Highlighting the defense and biotic and abiotic resistance functions shared by OH and IA-MN wild populations. Within the shared functional groups, responses to biotic and abiotic stressors were found to be overrepresented in wild populations from both OH and IA-MN.

Fig. S10 Gene co-expression network analysis. **(a)** Independent gene networks for both weedy and wild populations. **(b)** Overlapped gene detection for each gene module between weedy and wild samples. Each row of the table corresponds to one gene module for weedy samples and each column corresponds to one module for wild samples. Each number in the table indicates overlapped gene count between the same weedy and wild gene module. Fisher's exact test p-values in $-\log_{10}$ format were used to show significance for the overlap of the two modules. The stronger the red color, the more significant the overlap. **(c)** Conserved and variable gene modules with corresponding biological functions between weedy and wild samples.

Fig. S11 Genotype frequency of all SNPs across other herbicide target genes. For each herbicide resistant gene, the SNP across the entire genes were listed. For each SNP, the distribution of three different types of genotypes were calculated in the whole population.
